# Towards Large Diameter Transmit Coils for 7‐T Head Imaging: A Detailed Comparison of a Set of Transmit Element Design Concepts

**DOI:** 10.1002/nbm.70030

**Published:** 2025-04-05

**Authors:** Max Joris Hubmann, Stephan Orzada, Robert Kowal, Johannes Anton Grimm, Oliver Speck, Holger Maune

**Affiliations:** ^1^ Siemens Healthineers AG Erlangen Germany; ^2^ Faculty of Electrical Engineering and Information Technology Otto‐von‐Guericke University Magdeburg Germany; ^3^ German Cancer Research Center Heidelberg Germany; ^4^ Research Campus STIMULATE Magdeburg Germany; ^5^ Faculty of Physics and Astronomy Heidelberg University Heidelberg Germany; ^6^ Faculty of Natural Sciences Otto‐von‐Guericke University Magdeburg Germany

**Keywords:** 7 T MRI, antennas, EM simulations, hardware, head imaging, RF coils, RF shimming, UHF

## Abstract

Many different transmit (Tx) coil concepts and designs for 7‐T magnetic resonance imaging of the head have been proposed. Most of them are placed close to the head and in combination with the receive coils creating a helmet‐like structure. This limits the space for additional equipment for external stimuli. A large diameter transmit coil can increase the ease using supplementary measurement devices. Therefore, this study systematically evaluated nine different Tx elements regarding their performance within a large diameter transmit coil with a diameter > 350 mm. Each Tx element was examined regarding its power and specific absorption rate (SAR) efficiencies, its loading dependence, intrinsic decoupling, and its radio frequency (RF) shimming capability. Additionally, an experimental validation of $\left|B_{\text{1}}^{\text{+}}\right|$‐maps was performed. The loop‐based Tx elements (circular and rectangular loop) provided the highest power and SAR efficiency with at least 15.5% and 21.2% higher efficiencies for a single channel and 22.1% and 18.0% for the eight‐channel array, respectively. In terms of voxel‐wise power efficiency, the circular loop was the superior Tx element type within most of the head. Looking at the voxel‐wise SAR efficiency, the loop‐based elements manifest themselves as the most efficient type within most of the central brain. The mutual coupling was lowest for the passively fed dipole (− 31.23 dB). The highest RF shimming capability in terms of sum of normalized singular values was calculated for the rectangular (4.21) and the circular loop (4.36), whereby the L‐curve results showed that the arrays have only minor $\left|B_{\text{1}}^{\text{+}}\right|$ shimming performance differences for the transversal slice. For the hippocampus, the meander element provided the highest overall homogeneity with a minimal coefficient of variation (CoV) of 5.1%. This work provides extensive and unique data for single and eight‐channel Tx elements applying common performance benchmarks and enables further discourse on multi‐channel evaluations towards large diameter Tx coils at 7‐T head imaging. On the bases of the provided results, the preferable Tx element type for this specific application is loop‐based.

## Graphical abstract

This work compares nine different transmit element types towards their potential as large diameter (> 350mm) transmit head coils in single channel and eight channel array configurations. For most of the ROIs within the human head, the loop‐based elements were superior, and the RF shimming capability differed only marginally; however, in the deeper brain regions, dipole‐based elements gained in efficiency. For this particular application, the loop‐based transmit elements were superior to the remaining elements.

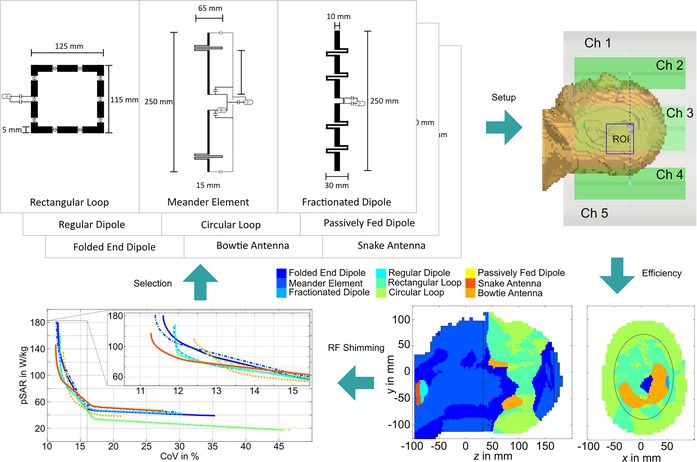

## 1 Introduction

In recent years, new frontiers have been set in various aspects of magnetic resonance imaging (MRI). Modern hardware components improved image acquisition [1, 2, 3, 4, 5, 6], and ultra‐high field (UHF) imaging with $B_{0}\geq 7\;T$ allowed a huge increase in image quality [7, 8, 9]. In the last decade, UHF imaging gained importance especially for neuro‐imaging [7, 10, 11, 12] due to these advances. Furthermore, some 7‐T systems have received clinical clearance in the United States and in the European Union. Thus, the first UHF scanners are routinely used in clinical settings [13]. To further advance neuro‐imaging using diffusion tensor imaging (DTI), the CONNECTOM project for 3 T has been initiated [14]. A new series of 7‐T scanners use their exceptional gradient strength and in combination with the high static magnetic field strength of 7 T will break boundaries down to the microscopic scale.

Despite several advantages, imaging at UHF comes with $\left|B_{\text{1}}^{\text{+}}\right|$‐field heterogeneities and specific absorption rate (SAR) constraints due to wavelength effects [9, 15]. These limitations result in reduced image quality or limited image acquisition capability. This can be addressed by substituting the classical transmit (Tx) coil architecture of a volume body coil with multiple smaller local Tx elements. These elements can be excited individually and are thereby combined to form a Tx coil array [16, 17]. The additional degrees of freedom create entirely new possibilities for image generation [18].

For UHF Tx elements, many different design concepts have been introduced [19, 20]. Some concepts rely on a dipole or monopole as the Tx element [21, 22, 23, 24, 25, 26]. Others, specifically for head imaging, are loop‐based [3, 5, 6, 27, 28]. Additionally, microstrip transmission line‐based Tx elements have been used for 7‐T Tx coils [29, 30]. Furthermore, more specialized concepts were introduced [31, 32, 33, 34]. Most of the designs target a specific application and shall be positioned close to the object. For head imaging, the radiofrequency (RF) Tx and receive (Rx) coils are placed as close to the head as possible to increase efficiency, thus generating a helmet like structure [3, 5].

This can increase the number of claustrophobic events and reduce space for external measurement equipment. In their cohort, Hudson et al. reported 0.76% of MRI examinations as incomplete due to claustrophobia related incidents [35]. Although received coils are positioned closer to the patient and can increase the claustrophobia incident rate themselves [35], particularly, the local RF shield of some UHF Tx coils shows the potential of increasing the number of claustrophobic incidents, since it is a closed structure. Head Tx coils, specifically engineered to reduce claustrophobia, sit tight around the head [3, 5], thereby restricting the space for supplementary measurement equipment. Incorporating devices such as EEG electrodes into conventional head coils typically necessitates additional modifications to the measurement hardware [36]. Designing a Tx coil with an increased diameter offers a solution by providing additional space, facilitating easier access to the head for delivering external stimuli (visual [37, 38, 39], auditory [40, 41], electric [42, 43], olfactory [44, 45], tactile [46]), to add further sensing equipment, such as for eye [47, 48] or motion tracking [49, 50, 51], or to place local receive elements. At the same time, the patient discomfort is reduced. Ideally, the Tx coils are placed behind the boreliner. Thus, the scanning routines are much more convenient as only receive coils must be placed on the subject, comparable to 1.5‐T and 3‐T systems.

The huge number of different Tx elements designed for diverse purposes, coupled with the numerous benchmarking methods available, complicates the selection of an appropriate Tx coil element based solely on a literature review. Moreover, the performance of different Tx element types in large‐diameter Tx coils, where the distance between the Tx elements and the target object increases, has not been thoroughly investigated. Consequently, selecting a suitable Tx element for this application based on existing studies remains challenging. A comprehensive list of benchmarks can be found in Choi et al. [52].

This work presents a detailed evaluation of a set of different Tx element concepts dedicated towards large diameter Tx arrays for UHF MRI of the head at 7 T. On the basis of a single‐ and multi‐element comparison using electromagnetic (EM) simulations and scanner field maps, the power, SAR efficiency, the mutual coupling, loading dependence, and power balance as well as the RF shimming capability were examined. Thereby, the compared Tx element concepts were taken from the literature but adapted, built, and simulated for the described application.

## 2 Methods

### 2.1 Transmit Elements Under Evaluation

**FIGURE 1:**
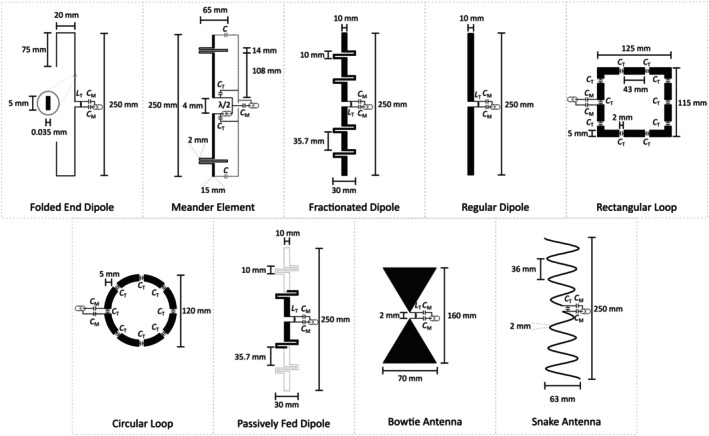
Examined transmit elements with their geometries and matching circuits: folded end dipole, meander element, fractionated dipole, regular dipole, rectangular loop, circular loop, passively fed dipole, bowtie antenna, and snake antenna [59, 60].

For this study, only Tx elements mentioned in the literature and designed for 7‐T imaging were evaluated. The following Tx elements were investigated: folded end dipole [21], meander element [53], fractionated dipole [54], regular dipole [55], rectangular loop [6], circular loop [56], passively fed dipole [26], bowtie antenna [57], and snake antenna [58]. The Tx elements are depicted in Figure 1. The elements were designed to fit into a circular array consisting of eight elements, constrained by a maximum element thickness of 20 mm due to geometrical limitations. The Tx element dimensions were adapted from their original designs but shortened to a maximum length of 250 mm. The width of the loop‐based Tx elements was calculated to fit a 1×8 cylindrical array with a diameter of 350 mm. In contrast, the dipole‐ and microstrip‐based Tx elements were not further shortened to ensure sufficient electrical length, which is necessary to limit the resulting conservative electric fields [54].

Each Tx element was tuned and matched to 50 Ω with the use of lumped elements, achieving input reflections $\left|S_{11}\right|$ below − 50 dB. The corresponding tuning and matching networks are depicted in Figure 1. Lumped element capacitors were modeled as first‐order parasitic models, with their equivalent series resistance extracted from the datasheet of the 2 kV non‐magnetic capacitor series by Knowles Syfer, USA (Serial Number for 6.2 pF: 111122K06P20BQTAF9LM). The lumped element inductors were modeled as parasitic Q‐models, with quality factors taken from the datasheets of the 1111SQ and 1515SQ air core inductor series provided by Coilcraft, USA. The numerical EM full‐wave simulations were carried out in CST Studio Suite 2022's (D'Assault Systémes, Vélizy‐Villacoublay, France) time domain solver. The co‐simulation was facilitated by employing the internal schematic simulator. The meshing strategy ensured that the substrate and all critical structural components contained at least two mesh cells per corresponding structural element.

### 2.2 Single Element Evaluation

#### 2.2.1 Loading Dependence

The impedance at the input port of a Tx element is influenced by both the element's intrinsic properties and the radiation resistance, which is itself dependent on the loading experienced by the Tx element. Thus, changes in the loading alter the radiation resistance and consequently, the impedance at the Tx element's input port [61, 62]. As described in section 2.1, the Tx elements were tuned and matched to 50Ω. Changing the loading will result in a mismatched, less efficient Tx element due to higher input reflections.

The intended application of the evaluated Tx elements is head imaging, where patient‐specific variations in head size are well documented [63, 64]. Consequently, the loading dependence of each Tx element is a key performance benchmark for a reliable Tx coil. The assessment was performed using simulations and measurements.

**FIGURE 2:**
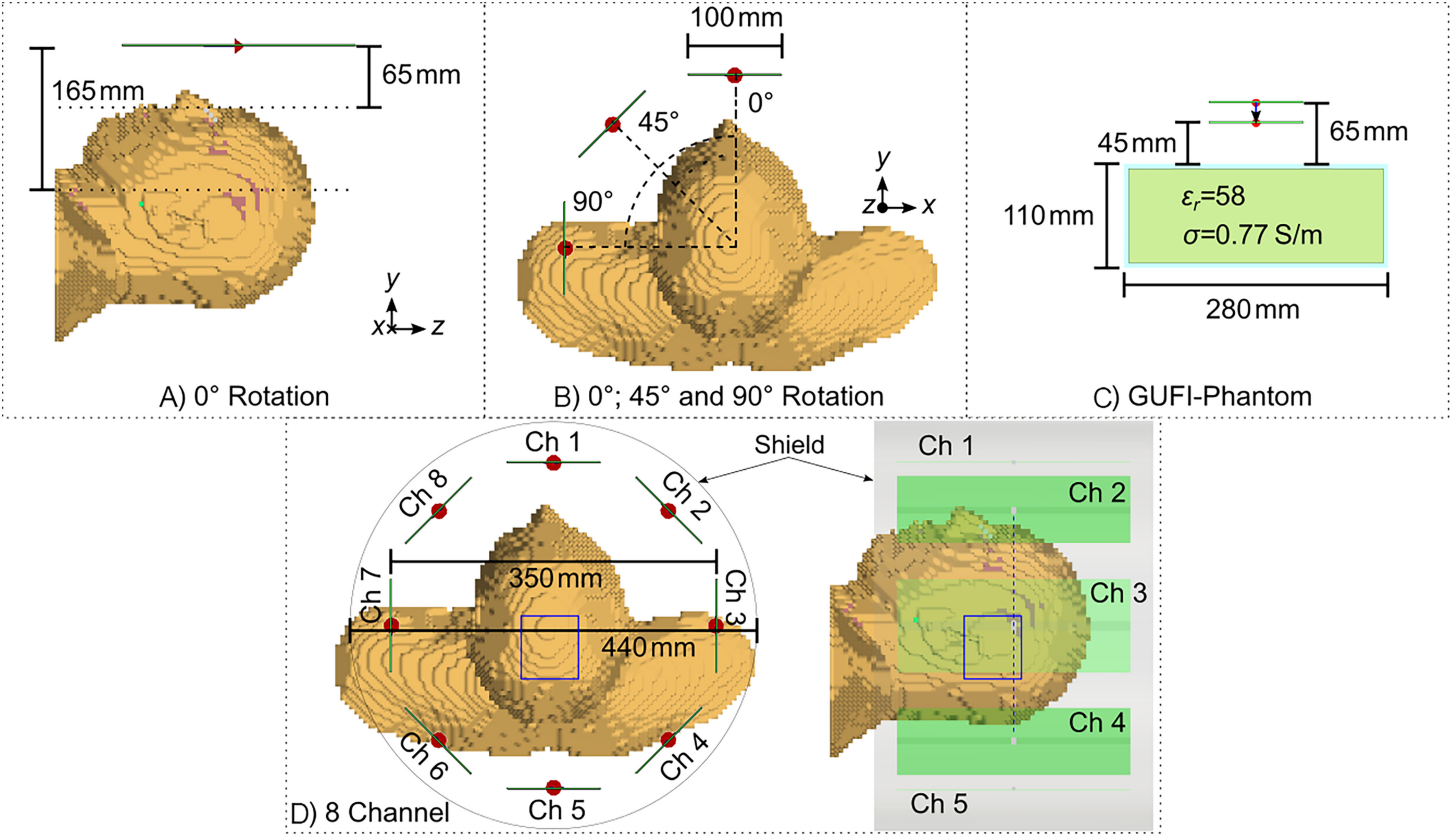
Simulation scenarios for the evaluation of the power and SAR efficiency for the single‐channel setup in (a)–(c). The Tx elements are marked as green lines with the feed port in red, exemplary visualized for the regular dipole. The dotted lines in (a) show the evaluation depths at 65 and 165 mm underneath the Tx element. In (b), the dashed lines show the positions 0°, 45°, and 90°, which were used to calculate the power and SAR efficiencies. The GUFI‐phantom [65] setup for the simulation and the measurement is depicted in (c). The eight element setup exemplary for the regular dipole is shown in (d) with the 440‐mm diameter shield [59]. The dotted blue line describes the position of the central transversal slice and the blue rectangle the position of the shim volume for the hippocampus [59, 60].

Each Tx element was positioned 65 mm above the GUFI‐phantom [65] and adjusted to achieve input reflections of less then − 20 dB. To emulate variations in loading, the distance between the phantom and the Tx element was decreased by 20 mm. This setup is depicted in Figure 2c. The change in $\left|S_{\text{11}}\right|$ resulting from this adjustment was determined by comparing the input reflection at the tuned state (− 20 dB) with the reflection observed at the reduced distance. This difference was expressed as a percentage, representing the reduction in accepted power due to the change in loading.

#### 2.2.2 Power Balance

The power balance provides information on how the power is distributed within Tx elements. Thus, it provides valuable information about the Tx elements' behavior. The nine Tx elements' power balance was evaluated for both the single element simulation and the eight‐channel array with a total stimulated power of 1 W employing the CST built‐in power loss calculation. It was thereby differentiated between the losses inside the lumped elements, the phantom, the substrate, and the radiation. Additionally, the losses due to coupling were included for the eight‐channel configurations. The reflected power was neglected in the calculations since the Tx elements were tuned to at least − 50 dB resulting in only marginal residual reflected power.

The eight‐channel array was excited in positive circular polarization (CP^+^) mode only. The CP^+^ mode is a commonly used excitation mode and provides valuable information about the power balance of the array configurations, although different excitation modes will result in different power distributions [66]. The CST power balance follows the logic described in [67].

#### 2.2.3 Power and SAR Efficiency

Since RF amplifier power is limited, to generate sufficient flip angles with short RF‐pulses, it is crucial that the Tx elements generate as much $\left|B_{\text{1}}^{\text{+}}\right|$‐field from the available power as possible [30, 68]. This power efficiency is the $\left|B_{\text{1}}^{\text{+}}\right|$‐field usually normalized to a stimulated input power of 1 kW or 1 W. Here, it is normalized to 1 W and represented by $|B_{\text{1}}^{\text{+}}|/\sqrt{P}$.

The SAR efficiency is based on the power efficiency combined with patient safety defined as the $\left|B_{\text{1}}^{\text{+}}\right|$‐field per square root of the peak local 10‐g SAR ($|B_{\text{1}}^{\text{+}}|/\sqrt{\text{pSAR}}$). Here, the SAR is a measure accounting for patient heating and limits the patient risk [69, 70].

To examine the power and SAR efficiency, the use‐case of a large diameter Tx coil was considered with a Tx element‐phantom‐distance between 50 and 80 mm. Therefore, the examination was performed in simulations at three positions with respect to the head and shoulders of the Hugo body model (resolution 5 mm×5 mm×5 mm). The rest of the body was removed to reduce computational effort. Since research showed that including the shoulders positively impacts the simulation accuracy, they were included [71]. For the first position, one single element was placed 65 mm above the head of the Hugo body model (see Figure 2a,b). The nose faced the Tx element but was neglected regarding the distance [59]. For the second and third positions, each Tx element was rotated by 45° and 90°, respectively. The setups are depicted in Figure 2b. For all three positions (0°, 45°, and 90°), the power and SAR efficiency 65 and 165 mm underneath the Tx elements were examined [59]. The SAR efficiency is based on the CST internal SAR calculation implementing IEC 62704‐1 [72].

#### 2.2.4 Phantom Validation

**FIGURE 3:**
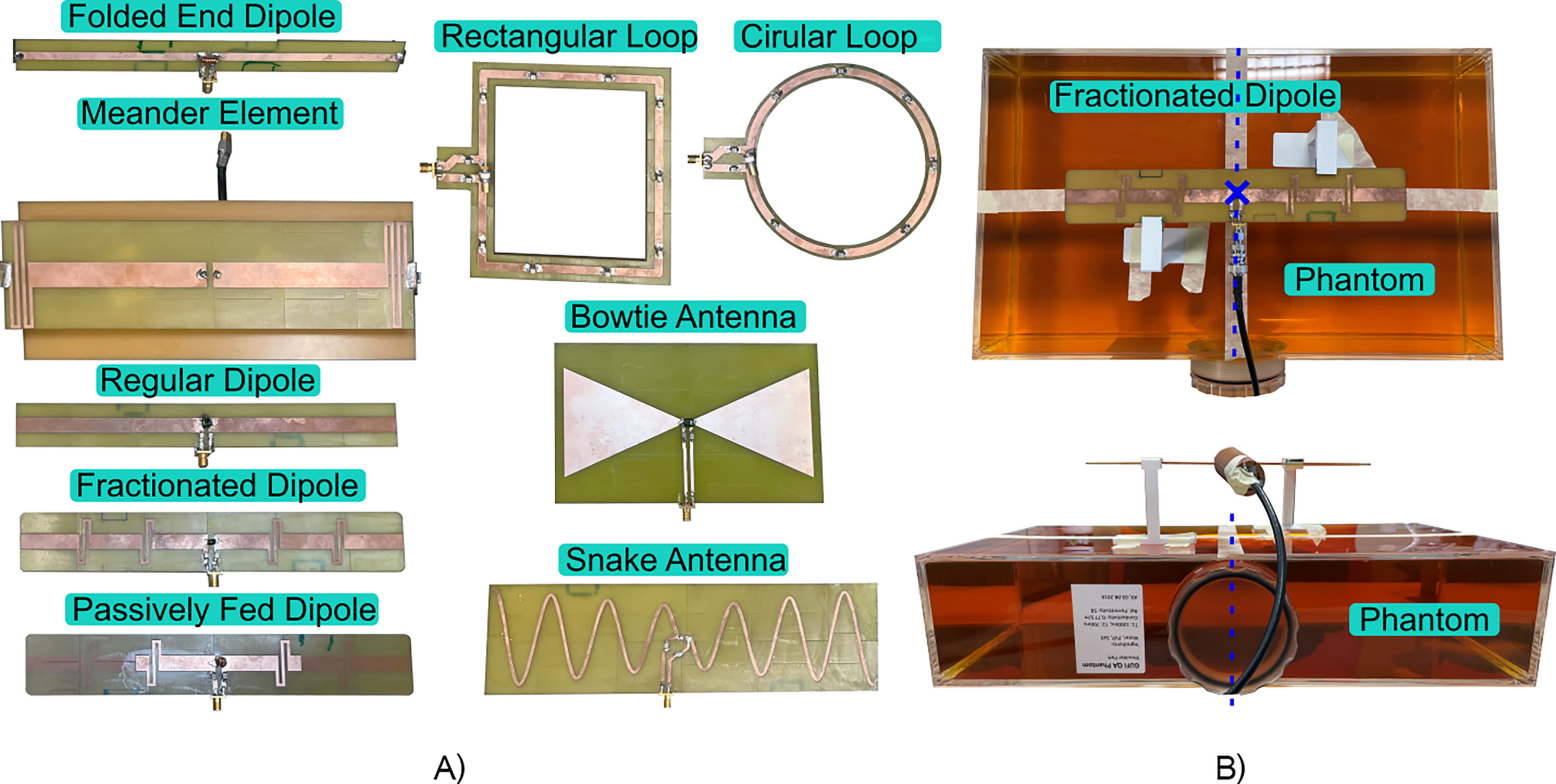
Constructed Tx elements (a) and the measurement setup for the $\left|B_{\text{1}}^{\text{+}}\right|$ measurement from the top (top) and from the side (bottom) exemplary for the fractionated dipole during the tuning and matching in (b).

To guarantee that the simulations reflect the real transmit elements, the described elements were built (see Figure 3a), and $\left|B_{\text{1}}^{\text{+}}\right|$‐maps were acquired to validate the simulations. Thereby, a fair comparison of the Tx elements was facilitated. For the validation, the Tx elements were placed 65 mm above the center of the GUFI‐Phantom [65] (see Figure 3b). Each element was tuned and matched on the bench to at least − 20 dB and connected to a MAGNETOM 7‐T prototype MRI scanner (Siemens Healthineers AG, Erlangen, Germany) via a transmit receive switch. With an actual flip angle imaging (AFI) sequence, two gradient echo images were acquired. The sequence parameters are depicted in Table 1. In post‐processing, the absolute flip angle was calculated based on Yarnykh [73]. A rectangular pulse shape was selected, so that finally, the $\left|B_{\text{1}}^{\text{+}}\right|$ normalized to 1 W was calculated using the following equation:

(1) $$|B_{\text{1}}^{\text{+}}|=\left(\text{11.7}\mu \text{T}\cdot \frac{\alpha_{\text{meas}}}{\alpha_{\text{nom}}}\right)/\sqrt{\frac{V_{\text{ref}}^{2}}{50\Omega }}$$

**TABLE 1:** AFI sequence parameters for the $\left|B_{\text{1}}^{\text{+}}\right|$‐map measurement.

Parameter	Sequence name	Slices per slab	Slabs	FoV read	FoV phase
Value	AFI	192	1	256 mm	50%

Parameter	Voxel size	TR1 | TR2	TE	Flip angle ($\alpha_{\text{nom}}$)	Base resolution
Value	2 mm×2 mm×2 mm	25ms | 125ms	2.04 ms	60°	128

Parameter	Bandwidth	Excitation	Dimension	Pulse length	RO | 3D spoiler factor
Value	490 Hz/Px	None‐sel. (rectangular pulse)	3D	1 ms	20 ms | 40 ms

Abbreviations: FoV = field of view; RO = readout; TE = echo time; TR = repetition time.

Here, 11.7 μ T is the $\left|B_{\text{1}}^{\text{+}}\right|$ necessary to generate a 180° flip angle using a 1ms long rectangular excitation pulse [61]. The measured and the nominal flip angle of the sequence are indicated by $\alpha_{\text{meas}}$ and $\alpha_{\text{nom}}$, respectively. The normalization to 1 W is performed by the denominator of the equation, whereby $V_{\text{ref}}$ is the reference voltage of the sequence. The calculated $\left|B_{\text{1}}^{\text{+}}\right|$‐maps were evaluated using a plane through the center of the phantom. The plane is indicated by the dotted blue line in Figure 3b.

After the measurement, the setup was modeled in CST including the attenuation of the transmission line (TxRx switch, cable trap, and feed line) from the scanner to the element (see Figure 2c). The measured attenuation was − 1.23 dB. Each Tx element was tuned to the previously measured absolute *S*‐parameters. The simulated $\left|B_{\text{1}}^{\text{+}}\right|$‐maps were calculated and compared to their measured equivalents.

### 2.3 Eight‐Channel Evaluation

To evaluate the described transmit elements in a multi‐channel arrangement, eight of each Tx element types were arranged equidistantly around a cylinder with a 350‐mm diameter. Again, the Hugo voxel model was used and placed centrally in the arrays. A shield with a 440‐mm diameter was placed around the elements. It mimics the global RF shield of the IMPULSE head gradients or can be seen as a local shield of the Tx arrays when used in MRI systems with 600‐mm bores. The setup is depicted in Figure 2d, exemplary for the regular dipole.

#### 2.3.1 Mutual Coupling

Particularly for dense multi‐channel coils, the mutual coupling between elements is an important figure of merit. It describes how much power is coupled from one Tx element to the adjacent elements. The mutual coupling is usually characterized by *S*‐parameter matrices [3, 21, 74].

It is important to keep the mutual coupling between adjacent elements is as low as possible, as the coupling decreases the power efficiency of the individual elements [75, 76]. If elements are insufficiently decoupled, further decoupling actions must be taken, which increases the complexity of the Tx coil [28] and introduces additional losses [76].

The benchmark for the mutual coupling of two elements is the crosstalk between the input ports $\left|S_{\text{xy}}\right|$ of the elements. $\left|S_{\text{21}}\right|$ describes the port interactions by providing information about the transferred power from the input port of element one to the port of element two.

To evaluate the mutual coupling, the same setup as described in Section 2.2.3 was used (Figure 2d). Thus, a center‐to‐center distance of 137.4 mm was created. All $\left|S_{\text{xy}}\right|$ were examined, whereby no further decoupling methods were applied to the elements which were tuneable without decoupling. Only the rectangular loop and the circular loop were not tuneable without decoupling; therefore, capacitive decoupling was used. Further descriptions of the decoupling method are presented in the supporting information.

#### 2.3.2 Power and SAR Efficiency

Similar to the single element evaluation, the power and SAR efficiencies were examined in simulation. Therefore, the Tx arrays were excited in CP^+^ mode and the maximum power and SAR efficiency, normalized to a total of 1‐W stimulated power, were evaluated. As for the single element comparison, the CST internal SAR calculation was used.

Additionally, the maximum voxel‐wise power and SAR efficiencies were calculated for a central axial, sagittal, and coronal slice. The power efficiencies were calculated summing up the $B_{\text{1}}^{\text{+}}$ distributions of all channels [76]. The voxel‐wise SAR efficiencies were estimated employing an optimization based on the fmincon solver in Matlab 2021a. Using 200 random starting shim vectors, the maximum possible SAR efficiency for each voxel in the corresponding slice was calculated. Both provide information about the performance of each Tx element within the different regions of the head.

For the optimizations, virtual observation points (VOPs) were used. They were calculated by using a compression followed by a post‐processing based on the algorithms described by Orzada et. al in [77]. For each Tx array, 500 ± 10% VOPs were calculated changing the maximum overestimation of the worst‐case 10‐g local SAR (wcSAR_10g_) accordingly. In addition, the actual relative errors between the SAR‐matrices and the resulting VOPs were calculated for one million random shims and the medians of the relative error were evaluated to quantify the quality of the VOPs. Furthermore, the wcSAR_10g_ was calculated for each array.

#### 2.3.3 RF Shimming Capability

As a measure for the RF shimming capability of the different Tx arrays, the sum of normalized singular values (SnSV) can be used to quantify the possible degrees of freedom [76, 78, 79]. The SnSV describes the amount of new information provided by each additional Tx element. Furthermore, the smallest normalized singular value provides information about how close the whole array is to the optimum and whether new elements will add further RF shimming capabilities. Stelter et. al defined a value of 0.1 as the threshold for a negligible increase in SnSV [67].

To calculate the SnSV, $B_{\text{1}}^{\text{+}}$‐maps for each channel were calculated, whereby each field is generated by one of the elements while the remaining elements were passive. The 3D‐$B_{\text{1}}^{\text{+}}$‐fields were mathematically rearranged into an 8 ×number of voxel array. A singular value decomposition was performed on this matrix. Finally, the resulting singular values were normalized to the maximum singular value and summed up to the SnSV [76, 78, 79], thereby providing information about the RF shimming capability of each array within the whole head.

To evaluate the shimming performance of the eight‐channel arrays, the Pareto front between the coefficient of variation (CoV(v)) as a measure for homogeneity and the pSAR(v) was calculated. For the SAR calculation, the same set of VOPs (VOP) as described in Section 2.3.2 was used. The calculation of the Pareto front was performed using Matlab 2021a and the fmincon solver applying 500 starting points for each of the 250 weighting factors λ. λ was logarithmically varied from zero to one. The following minimization problem with the cost function ϕ(v) was solved for each λ:

(2) $$\phi (\text{v})=\underset{\text{v}}{\min}(1-\lambda )\cdot \text{CoV}(\text{v})+\lambda \cdot \text{pSAR}(\text{v})$$

(3) $$s.t.CoV(\text{v})=\frac{\text{std}\left(\left|\text{B}\cdot \text{v}\right|\right)}{\text{mean}\left(\left|\text{B}\cdot \text{v}\right|\right)}$$

(4) $$\text{pSAR}(\text{v})=\max\left({\text{v}}^{\text{H}}\cdot \text{VOP}\cdot \text{v}\right)$$

(5) |v|≤1000W

(6) mean(|B·v|)=5.85μT

The column vector v describes the complex excitation vector. The $B_{1}^{+}$ distributions of each channel are reorganized into column vectors and concatenated to the matrix B. The nonlinear equality constraint was set to a resulting mean $\left|B_{\text{1}}^{\text{+}}\right|$ within the regions of interest (ROIs) of 5.85 μ T, which corresponds to a 90° flip angle using a 1‐ms long rectangular pulse. The linear constraint was set to a maximum power per channel of 1 kW. The ROIs for the optimizer were defined as the Hugo voxel models head at a central transversal slice and a cuboid volume covering the hippocampus. This is similar to the chosen L‐curve ROIs used in Fiedler et al. [80] and Stelter et al. [67]. The transversal slice is thereby one slice of the brain. The hippocampus is a deep area of the brain with a lot of ongoing research.

## 3 Results

### 3.1 Single Element Evaluation

#### 3.1.1 Loading Dependence

**FIGURE 4:**
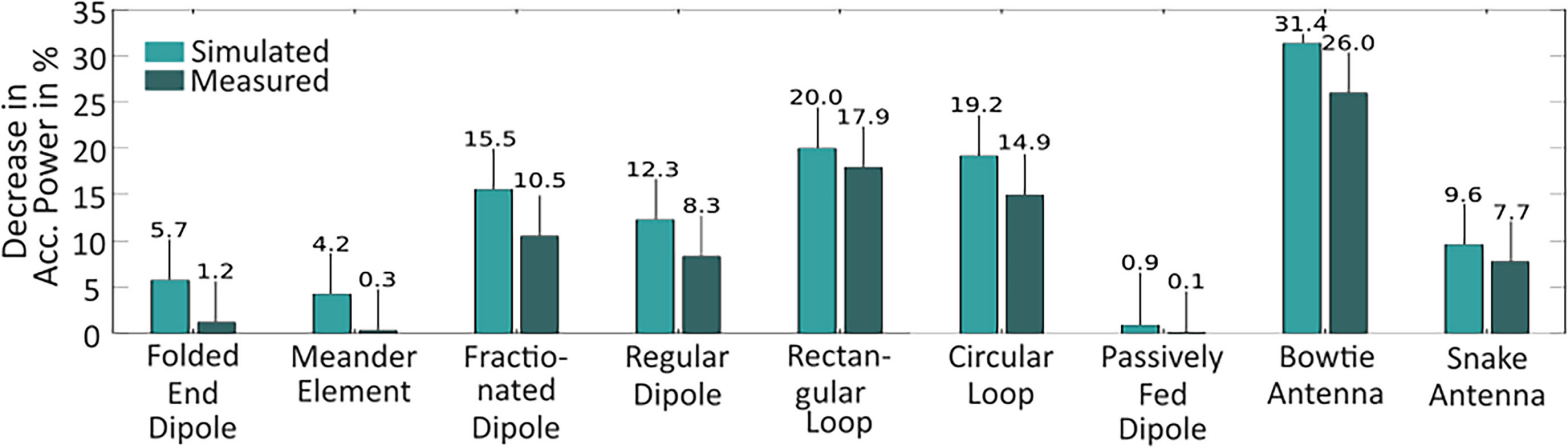
Measured (dark green) and simulated (light green) relative decrease in accepted power in % when changing the distance between the Tx element and the GUFI‐phantom from 65 to 45 mm.

The loading dependence was evaluated in simulation and in measurements using the GUFI‐phantom. The evaluation showed that there was a general qualitative agreement between the simulated and measured loading dependence. However, quantitatively, it is visible that the simulation generally overestimated the loading dependence by 2.1% to 5.5% (neglecting the passively fed dipole due to its very low total loading dependence). The bowtie antenna had the highest loading dependence with a decrease in accepted power by 31.4% for simulation and 26.4% for the measurement between the initial tuned state and the decrease in Tx element‐phantom distance. With 0.9% (sim.) and 0.1% (meas.), the passively fed dipole showed the least loading dependence, whereby the folded end dipole (5.7% and 1.2%) and the meander element (4.2% and 0.3%) also showed only low loading dependence. The results of the loading dependence evaluation are summarized in Figure 4.

#### 3.1.2 Power Balance

**FIGURE 5:**
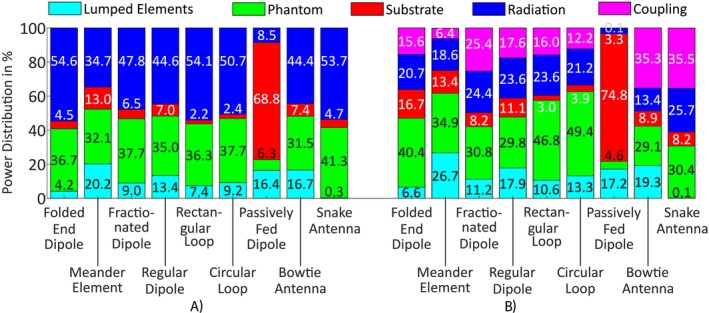
Single‐channel (a) and eight‐channel (b) power balance for the nine Tx elements normalized to 1‐W stimulated power.

The power distribution results for the single‐channel evaluation extracted from the CST simulation are depicted in Figure 5a and show that the elements induced between 31.5% and 41.3% of the input power to the head of the Hugo voxel model, except the passively fed dipole which provided much less power to the model (6.3%). At the same time, the passively fed dipole had the highest losses within the substrate, while radiating only 8.5% of the power. The highest radiation into the far field was generated by the folded end dipole with 54.6% and the least amount of lumped element losses occurred using the snake antenna.

#### 3.1.3 Power and SAR Efficiency

**FIGURE 6:**
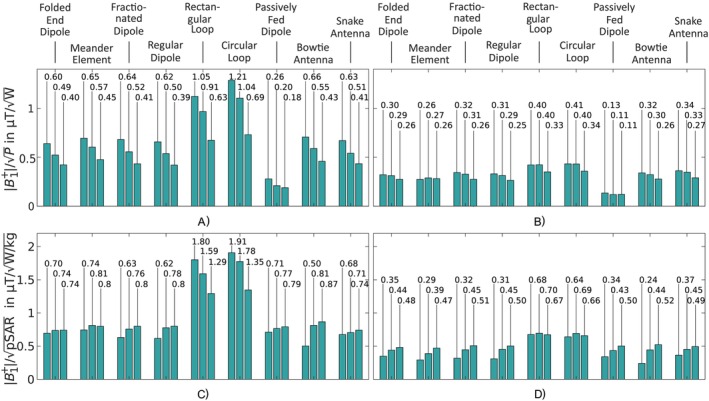
Maximum power (a and b) and SAR (c and d) efficiencies for the nine Tx elements for the three positions (0°, 45°, and 90°) at the surface of the head (a and c) and at 100 mm inside the head (b and d).

The power and SAR efficiency results were calculated using simulations with the head of the Hugo voxel model and are depicted in Figure 6.

The peak power efficiencies of the nine different Tx elements are summarized in Figure 6a,b. At 65 mm underneath the Tx elements at all three positions (0°, 45°, and 90°), the loop‐based Tx elements' rectangular loop and circular loop had the highest power efficiency with 1.05, 0.91, and 0.63 $\mu \text{T}/\sqrt{\text{W}}$ and 1.21, 1.04, and 0.69 $\mu \text{T}/\sqrt{\text{W}}$, respectively. The lowest power efficiency was shown by the passively fed dipole, which had only 21.5 % power efficiency of the circular loop for position 0°, 19.2% for position 45° and 18.8% for position 90°. All other Tx elements' power efficiencies were in between these boundaries, whereby the loop‐based Tx elements' efficiencies were at least 28.6% higher. All Tx elements showed the highest power efficiency for the 0° position and the lowest efficiency for the 90° position. At a distance of 165 mm, the most efficient Tx element was the circular loop with 0.40, 0.40, and 0.33 $\mu \text{T}/\sqrt{\text{W}}$ for the 0°, 45°, and 90° positions, respectively.

The passively fed dipole was the least efficient element with only 31.7%, 27.5%, and 33.3% compared to the circular loop at positions 0°, 45°, and 90°. In general, at a distance of 165 mm, the differences between the Tx elements were less significant, but the loop‐based Tx elements remained best with at least 15.5% higher power efficiency. Additionally, in difference to the evaluation at 65 mm, the highest power efficiency was not always shown at the 0° position.

The SAR efficiency results for Tx elements are depicted in Figure 6c,d. At a distance of 65 mm, all elements but the loop‐based elements and the meander element showed the highest SAR efficiency at position 90° and the lowest at 0°. The rectangular loop and circular loop had the highest SAR efficiency at position 0° and the lowest at position 90°, whereby the meander element peaked at 45°. In general, the dipole‐ and microstrip‐based structures provided similar SAR efficiency independent of the initial power efficiency. The overall highest SAR efficiency was generated by the circular loop with 1.91, 1.78, and 1.35 $\mu \text{T}/\sqrt{\text{W/kg}}$, whereby the rectangular loop showed only slightly less SAR efficiency. At position 0°, the lowest SAR efficiency with 0.50 $\mu \text{T}/\sqrt{\text{W/kg}}$ was generated by the bowtie antenna. At positions 45° and 90° with 0.71 and 0.74 $\mu \text{T}/\sqrt{\text{W/kg}}$, respectively, the snake antenna showed the lowest SAR efficiency. In general, the loop‐based Tx elements showed a at least 32.9% higher SAR efficiency than the remaining elements.

At a distance of 165 mm underneath the element, the highest SAR efficiency was again shown by the loop‐based Tx elements, whereby the remaining elements show only minor differences. The overall highest SAR efficiency was shown by the rectangular loop with 0.68, 0.70, and 0.67 $\mu \text{T}/\sqrt{\text{W/kg}}$ for all three positions. The lowest SAR efficiency for position 0° was provided by the bowtie antenna (0.24$\mu \text{T}/\sqrt{\text{W/kg}}$) and by the meander element for the remaining two positions (0.39 and 0.47 $\mu \text{T}/\sqrt{\text{W/kg}}$). Compared to the evaluation at 65 mm underneath the elements, with at least 21.2%, the difference in SAR efficiency between the loop‐based and the dipole as well as microstrip‐based elements was less significant.

#### 3.1.4 Phantom Validation

**FIGURE 7:**
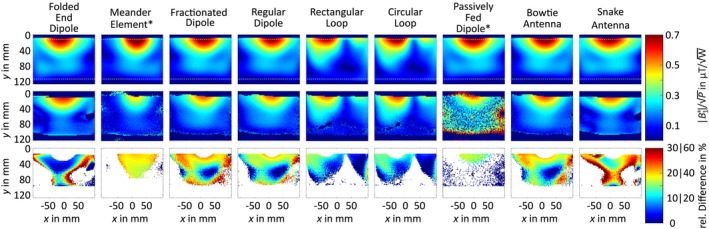
Simulated (1st row) versus measured (2nd row) $\left|B_{\text{1}}^{\text{+}}\right|$ distributions normalized to 1 W for the Tx elements; the dotted white rectangle describes the outline of the GUFI‐phantom; the 3rd row depicts the relative difference between simulation and measurement. *Note that the colormap for the passively fed dipole was scaled to 0.2 $\mu \text{T}/\sqrt{\text{W}}$ instead of 0.7 $\mu \text{T}/\sqrt{\text{W}}$ for better visualization and the scaling of the relative difference for the meander element and the passively fed dipole was increased from 0% to 60% for better visualization.

The comparison between simulated and measured $\left|B_{\text{1}}^{\text{+}}\right|$‐field distributions is depicted in Figure 7. The Tx elements show good qualitative agreement. However, quantitatively, there was a discrepancy between simulation and measurement. It was highest for the meander element and the passively fed dipole with more than 30% difference. For these two elements, there is a constant offset between measurement and simulation. For the other Tx elements, the difference was at maximum 10% to 25%, whereby the deviation between measurement and simulation was lower for the loop‐based elements as for the dipole‐based elements.

### 3.2 Eight‐Channel Evaluation

Some parts of the eight‐channel results were already published in the following abstract: Hubmann et al. [60].

#### 3.2.1 Mutual Coupling

**FIGURE 8:**
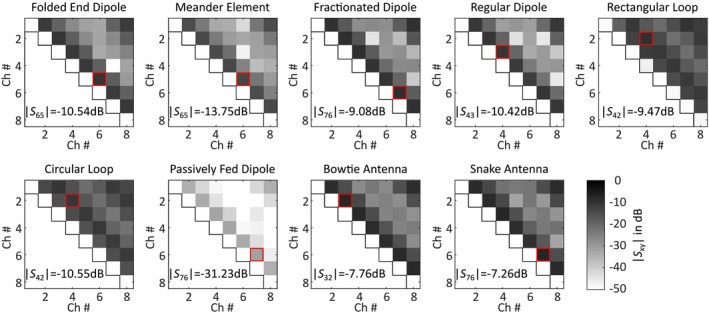
Intrinsic decoupling between the individual channels (Ch) of the nine Tx element arrays. The red rectangle marks the position with the highest mutual coupling. No additional decoupling was used except for the loop‐ based structures (rectangular loop, circular loop).

The simulated mutual coupling between the eight individual elements of the nine Tx arrays using the head of the Hugo voxel model is depicted in Figure 8. The strongest mutual coupling occurred for the snake antenna between channel (Ch) 6 and 7 ($|S_{\text{76}}|=-7.26$ dB). With a minimum of − 31.23 dB, the individual elements of the passively fed dipole interacted the least. For the dipole‐based arrays, the mutual coupling was highest between adjacent elements. For the loop‐based arrays, the next‐neighboring elements were the critical points. The reason was the capacitive decoupling of adjacent elements. For the loop‐based arrays, the worst‐case interaction is between the identical elements (Ch2 and Ch4), whereas the position of the worst‐case interactions varied for the dipole‐based arrays.

#### 3.2.2 Power Balance

The simulated power balance results for the eight‐channel arrays using the head of the Hugo voxel model are depicted in Figure 5b. The elements transferred 29.1% (bowtie antenna) to 49.4% (circular loop) of the incident power to the phantom; with only 4.6%, the passively fed dipole stands out as a notable exception. At the same time, the passively fed dipole array radiated the least into the farfield (3.3%) as well as having the lowest coupling losses (0.1%), while experiencing the highest losses in the substrate (74.8%). As for the single‐channel power balance, the highest and lowest lumped element losses were calculated for the meander element (20%) and the snake antenna (0.1%), respectively.

#### 3.2.3 Power and SAR Efficiency

**FIGURE 9:**
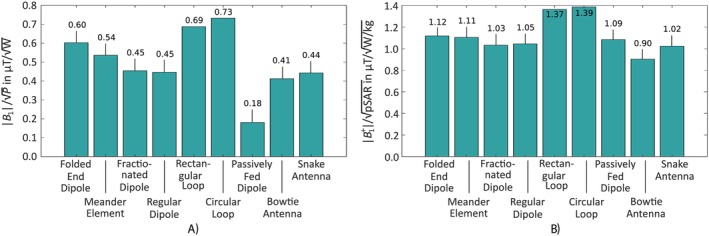
Maximum power efficiencies in (a) and maximum SAR efficiencies in (b) for the nine Tx element arrays exciting with a CP^+^ mode.

In the simulated CP^+^ mode, again, the loop‐based elements' circular loop and rectangular loop achieved the highest power efficiency (0.73 $\mu \text{T}/\sqrt{W/\mathrm{kg}}$ and 0.69 $\mu \text{T}/\sqrt{W/\mathrm{kg}}$, respectively), whereby the passively fed dipole with only 24.5% and 26.1% of the loop‐based elements', exhibited the lowest power efficiency. All other elements showed power efficiencies in between, and the meander element, fractionated dipole, regular dipole, bowtie antenna, and the snake antenna demonstrated similar values. The folded end dipole, however, with 0.60 $\mu \text{T}/\sqrt{\text{W/kg}}$ was more efficient. The simulation results are depicted in Figure 9a. Generally, excluding the folded end dipole, the loop‐based elements achieved at minimum a 22.1% higher power efficiency compared to the dipole‐based elements. Considering the Tx elements' SAR, the rectangular loop and the circular loop achieved the highest simulated efficiency (1.37 and 1.39 $\mu \text{T}/\sqrt{\text{W/kg}}$), whereby the lowest SAR efficiency was produced by the bowtie antenna (0.90 $\mu \text{T}/\sqrt{\text{W/kg}}$). The SAR efficiency results for the CP^+^ mode are depicted in Figure 9b. It is visible that the dipole‐based Tx elements all showed a similar SAR efficiency. The loop‐based elements thereby have a 18.0% to 33.8% higher SAR efficiency.

The necessary overestimations of the wcSAR_10g_ to attain the goal of 500±10% VOPs varied between 0.13% for the bowtie antenna and 1.35% for the circular loop. The calculation resulted in a maximum of 532 VOPs for the snake antenna and a minimum of 495 for the regular dipole. After post‐processing, the maximum relative overestimation for 1 million random shims ranged from 8.60% for the fractionated dipole to 14.57% for the passively fed dipole. The median of the relative overestimation calculated for 1 million random shims was in between 0.12% and 1.67% for the bowtie antenna and the circular loop, respectively. The detailed results are described in Table 2. With 2.30 W/kg the highest and with 0.09 W/kg the lowest wcSAR_10g_ were constituted by the bowtie antenna and the passively fed dipole, respectively. The remaining Tx arrays produced SAR levels in between.

**TABLE 2:** Used number of VOPs (# VOPs), resulting overestimation of the wcSAR_10g_ used for the VOP compression, maximum, and median of the relative overestimation of the final VOP sets calculated with 1 million random shims for the nine transmit elements, and wcSAR_10g_ for the nine Tx arrays.

	Folded end dipole	Meander element	Fractionated dipole	Regular dipole	Rectangular loop	Circular loop	Passively fed dipole	Bowtie antenna	Snake antenna
# VOPs	510	502	510	495	511	500	502	500	532
Maximum overestimation									
in % of wcSAR_10g_	0.55	0.55	0.21	0.22	1.20	1.35	0.50	0.13	0.31
Maximum relative overestimation									
% (after post‐processing)	9.73	15.58	8.60	9.99	13.34	12.72	14.36	8.64	10.36
Median relative overestimation in									
% (after post‐processing)	0.89	0.93	0.35	0.35	1.54	1.67	0.70	0.12	0.56
wcSAR_10g_									
in W/kg	1.24	0.95	1.96	1.64	0.99	1.04	0.09	2.3	1.42

**FIGURE 10:**
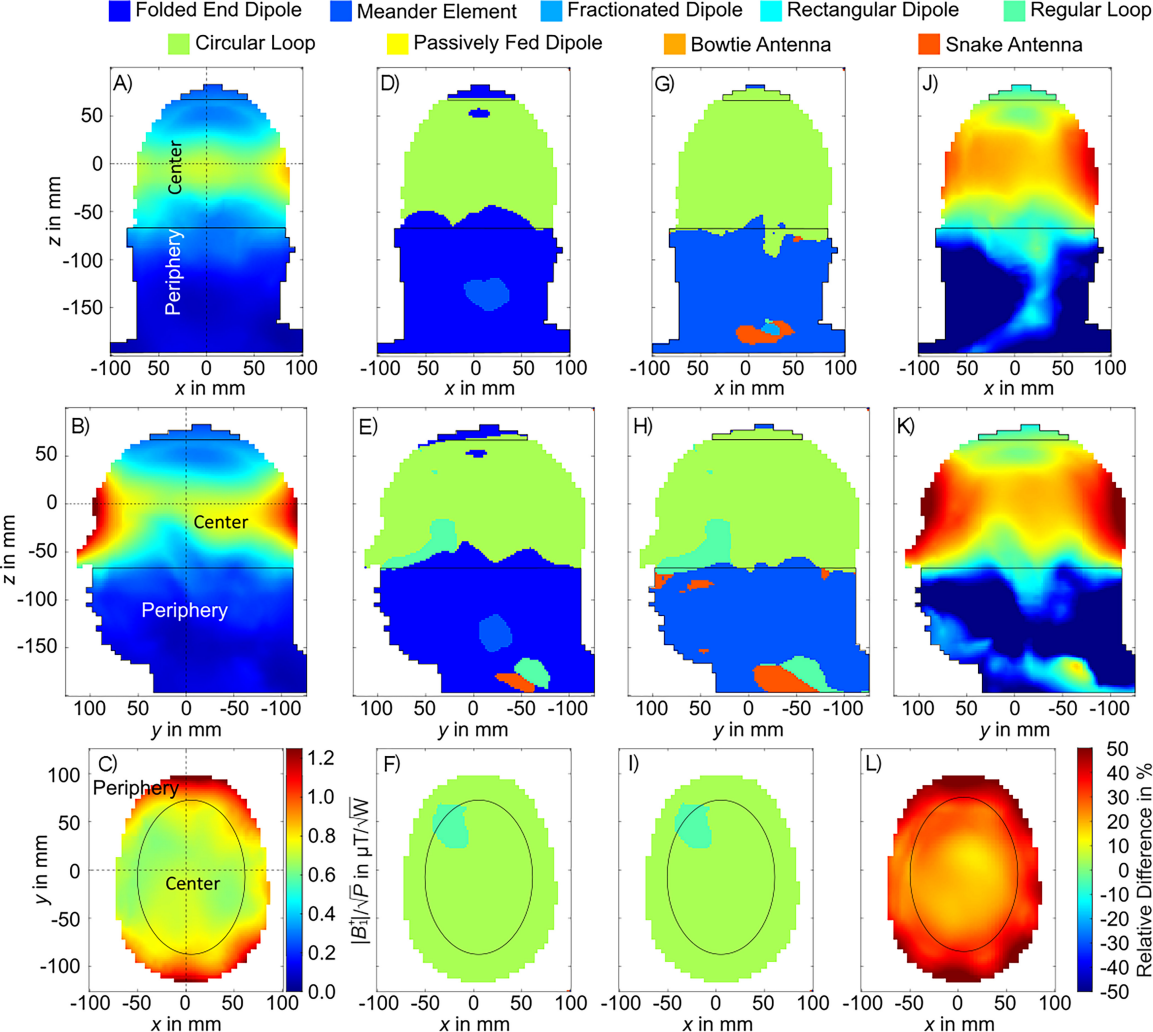
Voxel‐wise power efficiency as the added up $\left|B_{\text{1}}^{\text{+}}\right|$ of each channel of the circular loop for the central coronal (a), sagittal (b), and transversal slice (c). Voxel‐wise calculated most efficient Tx element out of the nine described elements for the same slices (coronal (d), sagittal (e), and transversal (f)) as well as without the folded end dipole (coronal (g), sagittal (h), transversal (i)). Relative difference between the most efficient element (circular loop) within the brain area and the most efficient element in the remaining area (folded end dipole) (coronal (j), sagittal (k), and transversal (l)). The dotted lines indicate the slice positions within the head and the solid lines differentiate between the slice selective defined peripheral and central areas. The center of each Tx element was positioned at z = 100 mm.

The simulated results of the voxel‐wise power efficiency using the head of the Hugo voxel model are depicted in Figure 10. In the peripheral transversal regions of the head (Figure 10c), a mean power efficiency of 0.85 $\mu \text{T}/\sqrt{\text{W}}$ could be achieved by the circular loop as the most efficient element. In the center, 0.71 $\mu \text{T}/\sqrt{\text{W}}$ was the maximum possible power efficiency, again generated by the circular loop. In the longitudinal periphery (Figure 10a,b), the folded end dipole was the superior element, especially towards the neck area. However, the folded part is close to the shield; thus, the shield influence was strong. Excluding the folded end dipole from the evaluation, a more diverse pattern of superiority for the longitudinal peripheral regions was revealed, whereby the meander element was superior in the majority of the area. In the transversal slice, the circular loop provided a 37.62% for the periphery and a 23.2% for the center higher power efficiency than the second most efficient Tx element (folded end dipole). In the sagittal and coronal slices for the majority of the brain, the power efficiency of the circular loop was higher than that of the folded end dipole, as indicated by a positive difference (Figure 10j,k). Towards the neck, the folded end dipole became more efficient resulting in negative differences. In the central region (see Figure 10j,k) the circular loop had an average of 16.5% higher power efficiency, whereby in the remaining area the folded end dipole had an average of 51.7% higher power efficiency.

**FIGURE 11:**
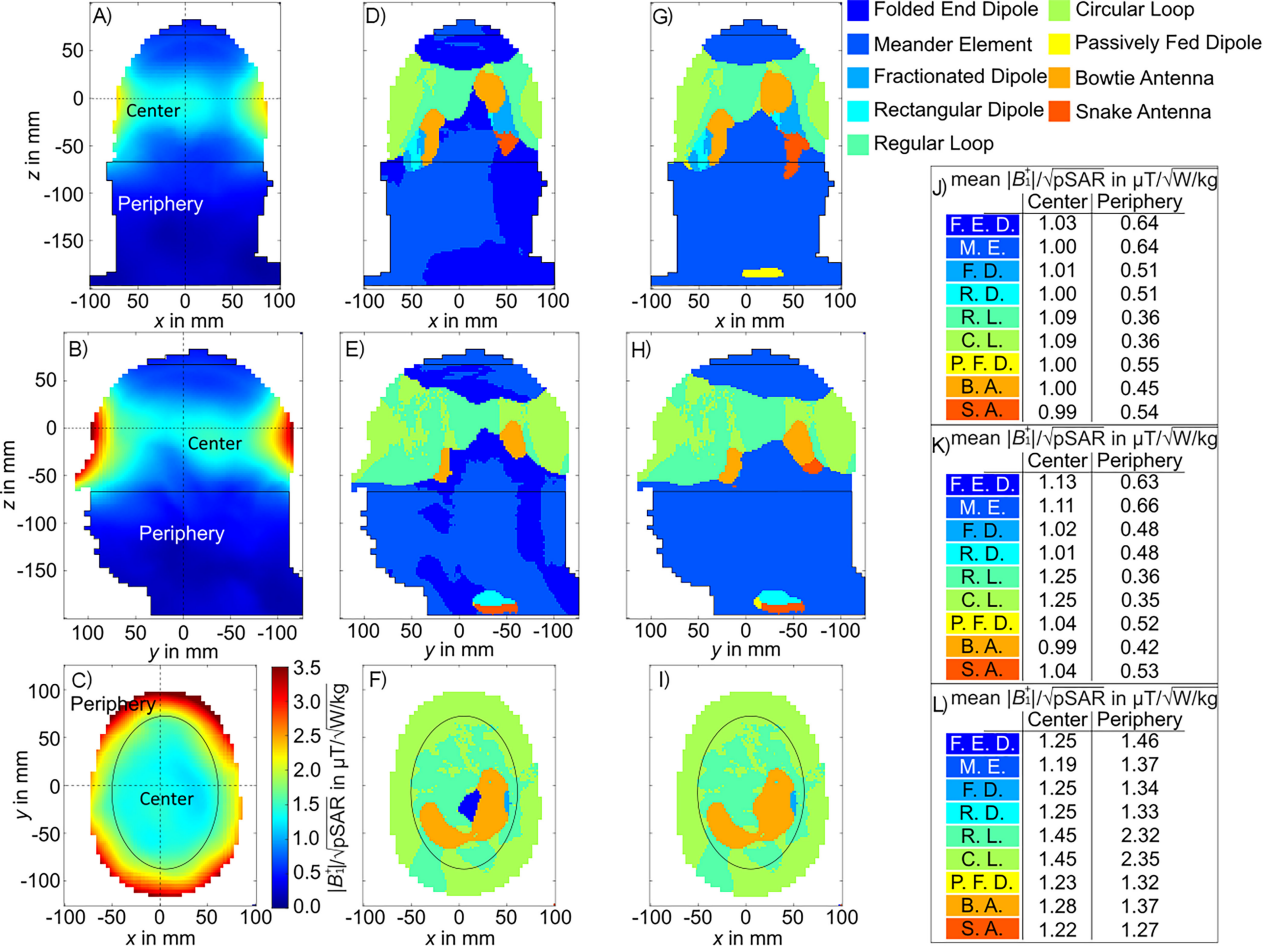
Simulated voxel‐wise SAR efficiency of the circular loop for the central coronal (a, d, g, j), sagittal (b, e, h, k), and transversal slice (c, f, i, l). Voxel‐wise calculated most SAR efficient Tx element out of the nine described elements for the slices (coronal (d), sagittal (e), and transversal (f)) and without the folded end dipole (coronal (g), sagittal (h), transversal (i)). Mean SAR efficiencies for the three slices (coronal (j), sagittal (k), and transversal (l)) for the corresponding central and peripheral regions. The dotted lines indicate the slice positions within the head and the solid lines differentiate between peripheral and central areas. The center of each Tx element is positioned at z = 100 mm.

The simulated results of the voxel‐wise SAR efficiency using the head of the Hugo voxel model are depicted in Figure 11. It is shown that the SAR efficiency decreased from the outside of head towards deeper regions (Figure 11a–c). In comparison to the power efficiency, there was no Tx element clearly superior to the other elements. For the majority of the central regions, the circular loop, the rectangular loop, or the bowtie antenna achieved the highest SAR efficiency. The peripheral regions, in transversal direction were dominated by the circular loop or the rectangular loop, whereby in most of the peripheral regions in sagittal and coronal direction, the folded end dipole or the meander element were dominant (Figure 11d–i). In the central region of the transversal slice, the circular loop and rectangular loop reached a mean SAR efficiency of 1.45 $\mu \text{T}/\sqrt{\text{W/kg}}$, which was the highest for all elements (Figure 11l) and corresponds to a 11.7% higher SAR efficiency compared to the next most efficient Tx array (bowtie antenna). In the sagittal slice, the superior elements for the central region were again the circular loop and the rectangular loop (1.25 $\mu \text{T}/\sqrt{\text{W/kg}}$). The same applies for the coronal slice (Figure 11g, 1.09 $\mu \text{T}/\sqrt{\text{W/kg}}$). For both directions, the achieved $|B_{\text{1}}^{\text{+}}|/\sqrt{\text{pSAR}}$ correspond to 4.8% and 9.8% higher SAR efficiency than the next most efficient Tx array, respectively. In the transversal periphery, the rectangular loop and circular loop reached a mean SAR efficiency of 2.32 and 2.35 $\mu \text{T}/\sqrt{\text{W/kg}}$, respectively, which was 37.2% and 38.0% higher compared to the 3rd most efficient Tx array (folded end dipole). In the sagittal periphery, the meander element achieved a mean SAR efficiency of 0.66 $\mu \text{T}/\sqrt{\text{W/kg}}$ providing 3.5% more $|B_{\text{1}}^{\text{+}}|/\sqrt{\text{pSAR}}$ as the 2nd (folded end dipole) and 19.9% more as the 3rd most effective Tx array. In the coronal periphery, the meander element as well as the folded end dipole stood out with the highest SAR efficiency by reaching 0.64 $\mu \text{T}/\sqrt{\text{W/kg}}$. This was 13.4% higher than the 2nd most efficient Tx array (passively fed dipole).

#### 3.2.4 RF Shimming Capability

**FIGURE 12:**
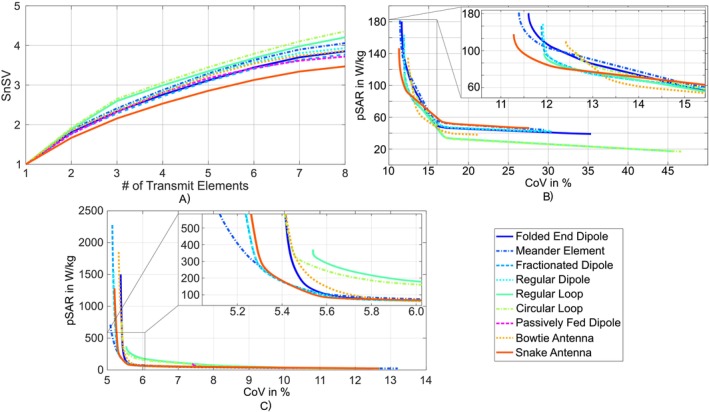
RF shimming performance. In (a), the sum of normalized singular values and the Pareto front for the nine Tx element arrays for the transversal slice (b) and for the hippocampus (c) as the ROI; the passively fed dipole could not achieve the 5.85μ T of mean $\left|B_{\text{1}}^{\text{+}}\right|$ within the ROI.

The results for the whole head of the Hugo voxel model of the SnSV evaluation are presented in Figure 12a. The loop‐based elements provided a higher SnSV over the whole course of elements and the difference between circular loop and rectangular loop and the other elements remained approximately constant. With a SnSV of 4.36 and 4.21, respectively, for the total array with eight elements, the circular loop and rectangular loop had the highest SnSV and thereby provided the most degrees of freedom. The strongest limitation in RF shimming performance was shown by the snake antenna with an SnSV of 3.47. The smallest minimal normalized singular value was 0.11 for the passively fed dipole and the highest was 0.25 for the circular loop. This demonstrates that none of the Tx element arrays reached its optimal array count. Particularly, for the circular loop and rectangular loop, additional elements could further increase the RF shimming performance. However, due to geometrical constraints, the loops must then be reduced in size.

The results of the Pareto front examinations for the RF shimming capabilities within the defined ROIs of the Hugo voxel models head are depicted in Figure 12b and c as L‐curves. For the transversal slice, aiming for a mean $\left|B_{\text{1}}^{\text{+}}\right|$ of 5.85 μ T, the minimum CoV was between 11.28% for the snake antenna and 12.64% for the circular loop. Generally, for a central transversal slice, all elements showed only minor differences in terms of homogeneity. For higher CoV (> 16%), the circular loop and rectangular loop generated a lower maximum pSAR compared to the remaining arrays.

For the hippocampus, which is a deep brain region close to the center of the head, the meander element provided the overall lowest minimum CoV (5.1%), while generating the a pSAR of 711 W/kg. The highest minimum CoV was generated by the rectangular loop with 5.5% generating 371 W/kg in pSAR. For strict SAR constraints the meander element provided the best trade‐off between SAR and CoV, as it provided the lowest pSAR for most of the CoVs. With the passively fed dipole, a mean $\left|B_{\text{1}}^{\text{+}}\right|$ of 5.85μ T could not be generated for most of the shim vectors at both of the ROIs due to the power constraints.

## 4 Discussion

In this work, nine different Tx element types for a 7‐T large diameter Tx head coil were evaluated in single‐ and eight‐channel configurations based on the power and SAR efficiencies, the mutual coupling, the loading dependence and the RF shimming capability using EM simulations. Furthermore, the results were validated using experimentally measured $\left|B_{\text{1}}^{\text{+}}\right|$‐maps.

The loading dependence results showed good agreement between simulation and measurement with the built Tx elements having a lower loading dependence compared to the simulated elements and the bowtie antenna was the element with the strongest loading dependence.

The power balance results showed that for single elements, all the Tx types transferred a similar amount of power to the phantom (31.5%–37.7%). Exceptions were the passively fed dipole with a lot less (6.3%) and the snake antenna with slightly more (41.3%) power into the phantom. For the eight‐channel configuration, the loop‐based elements transfer more power into the phantom than the remaining elements (46.8 % and 49.4 %). The mutual coupling results revealed that the snake antenna had the highest mutual coupling ($|S_{76}|=-7.26$ dB) and that the passively fed dipole coupled the least ($|S_{76}|=-31.23$ dB).

The single element comparison revealed that the loop‐based Tx elements (circular loop and rectangular loop) provided the highest power and SAR efficiency with at least 15.5% and 21.2% larger efficiencies, respectively. This holds for the eight‐channel arrays in the CP^+^ mode, where the loop‐based elements showed at least 22.1% higher power and 18.0% higher SAR efficiency.

In terms of voxel‐wise power efficiency the circular loop was the superior Tx element type within most of the head. Only towards the neck area, the folded end dipole became the best element. Looking at the voxel‐wise SAR efficiency, the loop‐based elements manifest themselves as the most efficient type within most of the central brain area whereby in the periphery the folded end dipole and the meander element were superior. Within all the defined central areas, the loop‐based elements had the highest mean SAR efficiency as well as power efficiency.

The evaluation of the RF shimming performance uncovered, that for the whole head, the calculated SnSV for the rectangular loop (4.21) and the circular loop (4.36) were highest whereby the L‐curve results showed that the arrays have only minor shimming performance differences for the transversal slice and the hippocampus. However, the loop‐based elements needed less SAR for CoVs > 16% aiming for a central transversal slice. For the hippocampus, the meander element provided the highest overall CoV and showed the best trade‐off between it and the pSAR.

The comparison between the measured and the simulated $\left|B_{\text{1}}^{\text{+}}\right|$‐maps showed good qualitative agreement; however, quantitatively, there was an offset between measurement and simulation.

Many different decoupling methods for loop‐based Tx elements were introduced [19, 28, 81, 82, 83, 84], whereby here the capacitive decoupling was chosen for simplicity. Elements with $|S_{\text{21}}|>-10$ dB generally require external decoupling. Therefore, the fractionated dipole, the bowtie antenna, and the snake antenna might also need further decoupling. However, this was not implemented for the presented arrays, since they were tunable without further decoupling. For these types of Tx elements, different decoupling methods were developed [30, 85, 86, 87] as well. Consequently, the need for additional decoupling in loop‐based Tx element arrays is not necessarily a drawback. However, the rectangular loop might need further decoupling of the next‐neighboring elements as performed in [5]. The Tx elements with higher coupling coefficients provided stronger losses due to coupling and vice versa, hence the decoupling results corresponded well to the calculated coupling losses.

For this setup, the loop‐based elements turned out to be the superior elements. Thus, considering the dimensions of the human head phantom in use, the Larmor frequency, and the head‐element distance, the advantages of dipole‐based elements within deeper regions [88] do not yet come into play. Additionally, the classically used dipole‐ and microstrip‐based Tx elements (folded end dipole, meander element, fractionated dipole, regular dipole, bowtie antenna, and snake antenna) exhibited similar power and SAR efficiency. Hence, for Tx elements used at a distance to the subject larger than 65mm, actions to reduce the conservative electric fields, like using meanders [22] or special dielectrics [32, 89, 90] are unnecessary. The passively fed dipole showed less efficiency compared to prior reports [26] showed. However, this can be explained by the different phantom‐element distance, which impacts its performance. The power efficiency results using the GUFI‐phantom showed only a minor difference between the Tx elements (except the passively fed dipole), which contradicts the findings using the Hugo phantom. As Figure S4 shows, this variation can be explained by the different phantom geometries. The voxel‐wise power and SAR efficiencies were higher in the periphery compared to the center. This is reasonable, since higher $\left|B_{\text{1}}^{\text{+}}\right|$ in the center necessitates increased input power, thus raising the pSAR. This is consistent with previously published work [67]. Interestingly, in certain areas of the brain, the bowtie antenna array showed the highest voxel‐wise SAR efficiency, although it showed the lowest SAR efficiency in CP^+^. This can be explained by the varying locations of the pSAR for different shim vectors [6].

Fiedler et al. [80] stated that transmit arrays with a high wcSAR_10g_ can be overestimated as stronger; thus, for arrays with a higher wcSAR_10g_, a lower overestimation factor can be used in order to generate an equal amount of VOPs. This was confirmed by the presented data.

The RF shimming results were only obtained for a transversal slice and a cuboid volume covering the hippocampus. For other regions, for example, the whole brain, different results can be expected. Generally, for larger ROIs, the maximum achievable homogeneity decreases.

None of the included elements were optimized for this application, and some of them were even designed for completely different setups and body regions. Still, the presented results lead to the conclusion that even with adapting and optimizing the presented elements, the loop‐based elements are likely to demonstrate superior performance compared to the other tested elements.

The deviations between the measured and simulated $\left|B_{\text{1}}^{\text{+}}\right|$‐maps were within comparable validation accuracies [3, 6, 54, 55, 74]. However, a deviation between measurement and simulation remained, representing an ongoing challenge within the scientific community. Some research groups introduce a scaling factor as proposed by Schmidt et al. [91] or adjust the Q‐factors of the elements. The assessment of loss mechanisms within Tx elements is ongoing research and is beyond the scope of this study.

The difference between measured and simulated loading dependence arose from several factors. First, the measurement was prone to measurement errors and influenced by the environment surrounding the coils. The measurement was not performed in a truly absorbing environment, thus having slightly different boundary conditions than the simulation. Second, the remaining difference between measurement and simulation indicated additional losses inside the Tx elements, which are not yet fully understood [91]. These losses can reduce the interaction with the loading and thereby explain the slightly lower loading dependence observed in the measurements.

The single‐channel evaluations were conducted without an RF shield, whereas the eight‐channel simulations incorporated a shield. The presence of this shield significantly reduced radiation losses. However, the varying thicknesses of the Tx elements resulted in some being positioned closer to the shield than others, which caused stronger interactions and altered their performance. This effect was particularly evident for the folded end dipole, where the folded ends formed a capacitor with the shield. This interaction affected the current distribution, thereby influencing both the power and SAR efficiencies [92]. The loop‐based Tx elements demonstrated substantially higher power and SAR efficiencies in both single‐channel and multi‐channel configurations. This indicates that single‐channel evaluations can provide meaningful insights into multi‐channel performance. Given that single‐channel simulations are significantly faster, they can expedite the design and optimization process for new Tx elements. The geometrical dimensions of the Tx elements varied, with loop‐based elements being notably smaller in the z‐direction. This accounted for the folded end dipole's superior performance in the neck region. However, within the brain, the chosen loop sizes of 125 mm × 115 mm for the rectangular loop and 120 mm for the circular loop performed superior. As shown in Figure S5, a large rectangular loop would still perform superior to the dipole‐based elements. Further reducing the dipole length was not considered in this work.

Inaccuracies in the measurement results of the $\left|B_{\text{1}}^{\text{+}}\right|$‐maps of the validation appeared due to gradient nonlinearity and drop‐outs from the receive performance. The higher deviation at high and low $\left|B_{\text{1}}^{\text{+}}\right|$ values were related to the limited dynamic range of the AFI sequence [73]. Furthermore, the GUFI phantom, which aged 6 years, may have experienced material property alterations, leading to inaccuracies in the assumed parameters [65]. Thus, the assumed material properties might not be accurate anymore [65]. Additionally, discrepancies between simulated and measured results were influenced by losses in the constructed Tx elements that differed from those assumed in simulations. This is highlighted by the similar accuracies observed among comparable elements (e.g., circular and rectangular loops or fractionated and regular dipoles). The greater deviation in dipole‐based elements likely originated from additional losses within the self‐wound tuning inductors, as the simulations relied on estimated losses derived from datasheets.

The power distribution results revealed significant power losses in the substrate of the passively fed dipole. The used substrate was FR‐4, which has inconsistent properties due to the manufacturing variability. This inconsistency is the probable source of the higher error for this element. For the meander element, the high deviation between measurement and simulation is most likely a combination of different effects, which are currently undergoing further evaluation. But the deviation is almost constant over the measurable area; hence, the deviation most likely originates in additional losses inside the built meander element which the simulation did not account for. However, further assessing the loss mechanisms is not within the scope of this work. Additionally, neither the meander element nor the passively fed dipole turned out to be the superior Tx element type; thus, the deviation between measurement and simulation does not significantly affect the final conclusion of this work.

The loop‐based Tx elements demonstrated a higher maximum SnSV, suggesting greater flexibility in sequence development as power and SAR constraints are less likely to be exceeded. However, the L‐curve results provide a contrasting perspective due to the lower CoV observed in the loop‐based elements. This discrepancy arises because the optimization focused on a central transversal slice and the hippocampus, whereas the SnSV was computed for the entire brain. Additionally, the SnSV metric exclusively considered $B_{\text{1}}^{\text{+}}$ distributions, neglecting SAR, while the L‐curve incorporated both $\left|B_{\text{1}}^{\text{+}}\right|$ and SAR into its calculations.

The passively fed dipole was unable to achieve the defined 5.85 μ T mean $\left|B_{\text{1}}^{\text{+}}\right|$ within the target ROIs, rendering it unsuitable for this application.

The evaluation of the minimal relative singular value indicated that none of the Tx elements reached the optimal element count for this specific setup. Geometrical constraints prevent the addition of more elements within a single ring for the loop‐based and meander elements. These limitations could be addressed by using a wider diameter or smaller elements, but such modifications would alter the overall arrangement and impact the results.

It is important to note that the Tx elements varied in their manufacturing and handling complexities. Loop‐based elements required careful attention to the lumped components, while the passively fed dipole necessitated precise consideration of the substrate material and geometric arrangement. The handling of the meander element was much more convenient compared to the other elements, since it was insensitive to materials behind the ground plane. However, for the next generation of MRI scanners, due to the strong gradients, actions to avoid eddy currents must be considered.

In this study, none of the Tx elements were specifically optimized for use in a large‐diameter Tx head coil. Future research should focus on optimizing these elements to enhance performance for the described application.

## 5 Conclusions

This contribution provides a detailed comparison of different Tx elements for 7‐T head imaging using EM full‐wave simulations considering the application in a large diameter Tx head coil resembling a conventional body volume coil. The comparison was validated using $B_{\text{1}}^{\text{+}}$ measurements on a 7‐T MRI scanner. It was demonstrated that the highest power and SAR efficiencies were generated by the circular and rectangular loop, whereby the highest intrinsic decoupling was shown by the passively fed dipole. Additionally, the value of single element simulations was proven. In simulation as well as in measurement, the loading dependence was strongest for the bowtie antenna. The validation showed comparable agreement between measurement and simulation indicating proper coil construction as well as simulation. Considering the available decoupling methods and the results, head imaging at 7 T using a Tx array with an inner diameter larger than 350 mm for better patient comfort and more space for auxiliary material, loop‐based Tx elements might be the preferred option. This work provides extensive and unique data for single‐ and eight‐channel Tx elements applying common measures and enables further discourse on multi‐channel evaluations towards large diameter Tx coils at 7‐T head imaging.

## Conflicts of Interest

The first author, Max Joris Hubmann, was an employee of Siemens Healthineers AG.

## Supporting information

Supporting_Information.pdf

## Data Availability

The data as well as all simulation files that support the findings of this study are available from the corresponding author Max Joris Hubmann upon request.

## References

[1] T. S. V. Gomez , M. Dubois , K. Rustomji , et al., “Hilbert Fractal Inspired Dipoles for Passive RF Shimming in Ultra‐High Field MRI,” Photonics and Nanostructures ‐ Fundamentals and Applications 48 (2022): 100988, 10.1016/j.photonics.2021.100988.

[2] N. Gudino and S. Littin , “Advancements in Gradient System Performance for Clinical and Research MRI,” Journal of Magnetic Resonance Imaging 57, no. 1 (2023): 57–70, 10.1002/jmri.28421. 36073722

[3] M. W. May , S. L. J. D. Hansen , M. Mahmutovic , et al., “A Patient‐Friendly 16‐Channel Transmit/64‐Channel Receive Coil Array for Combined Head–Neck MRI at 7 Tesla,” Magnetic Resonance in Medicine 88, no. 88 (2022): 1419–1433, 10.1002/mrm.29288. 35605167 PMC9675905

[4] K. Uğurbil , E. Auerbach , S. Moeller , et al., “Brain Imaging With Improved Acceleration and SNR at 7 Tesla Obtained With 64‐Channel Receive Array,” Magnetic Resonance in Medicine 82, no. 1 (2019): 495–509, 10.1002/mrm.27695. 30803023 PMC6491243

[5] S. N. Williams , S. Allwood‐Spiers , P. McElhinney , et al., “A Nested Eight‐Channel Transmit Array With Open‐Face Concept for Human Brain Imaging at 7 Tesla,” Frontiers in Physics 9 (2021): 412, 10.3389/fphy.2021.701330.

[6] S. N. Williams , P. McElhinney , and S. Gunamony , “Ultra‐High Field MRI: Parallel‐Transmit Arrays and RF Pulse Design,” Physics in Medicine and Biology 68(2):02TR02 (2023), 10.1088/1361-6560/aca4b7. 36410046

[7] G. Barisano , F. Sepehrband , S. Ma , et al., “Clinical 7 T MRI: Are We There Yet? A Review About Magnetic Resonance Imaging at Ultra‐High Field,” British Journal of Radiology 92, no. 1094 (2019): 20180492, 10.1259/bjr.20180492. 30359093 PMC6404849

[8] C. Le Ster , A. Grant , P. F. van de Moortele , et al., “Magnetic Field Strength Dependent SNR Gain at the Center of a Spherical Phantom and up to 11.7T,” Magnetic Resonance in Medicine 88, no. 5 (2022): 2131–2138, 10.1002/mrm.29391. 35849739 PMC9420790

[9] R. Pohmann , O. Speck , and K. Scheffler , “Signal‐to‐Noise Ratio and MR Tissue Parameters in Human Brain Imaging at 3, 7, and 9.4 Tesla Using Current Receive Coil Arrays,” Magnetic Resonance in Medicine 75, no. 2 (2016): 801–809, 10.1002/mrm.25677. 25820458

[10] D. A. Ciantis , C. Barba , L. Tassi , et al., “7T MRI in Focal Epilepsy With Unrevealing Conventional Field Strength Imaging,” Epilepsia 57, no. 3 (2016): 445–454, 10.1111/epi.13313. 26778405

[11] M. Filippi and M. A. Rocca , “MR Imaging of Multiple Sclerosis,” Radiology 259, no. 3 (2011): 659–681, 10.1148/radiol.11101362. 21602503

[12] Y. Ge , V. M. Zohrabian , and R. I. Grossman , “7‐Tesla Magnetic Resonance Imaging: New Vision of Microvascular Abnormalities in Multiple Sclerosis,” Archives of Neurology 65, no. 6 (2008): 812–816, 10.1001/archneur.65.6.812. 18541803 PMC2579786

[13] R. Voelker , “Twice the Power in New MRI,” Journal of the American Medical Association 318, no. 19 (2017): 1858, 10.1001/jama.2017.17120.

[14] S. Y. Huang , T. Witzel , B. Keil , et al., “Connectome 2.0: Developing the Next‐Generation Ultra‐High Gradient Strength Human MRI Scanner for Bridging Studies of the Micro‐, Meso‐ and Macro‐Connectome,” NeuroImage 243 (2021): 118530, 10.1016/j.neuroimage.2021.118530. 34464739 PMC8863543

[15] C. M. Collins and M. B. Smith , “Signal‐to‐Noise Ratio and Absorbed Power as Functions of Main Magnetic Field Strength, and Definition of 90 Degrees RF Pulse for the Head in the Birdcage Coil,” Magnetic Resonance in Medicine 45, no. 4 (2001): 684–691, 10.1002/mrm.1091. 11283997

[16] C. Wang and G. X. Shen , “B1 Field, SAR, and SNR Comparisons for Birdcage, TEM, and Microstrip Coils at 7T,” Journal of Magnetic Resonance Imaging : JMRI. 24, no. 2 (2006): 439–443, 10.1002/jmri.20635. 16786582

[17] A. G. Webb and C. M. Collins , “Parallel Transmit and Receive Technology in High‐Field Magnetic Resonance Neuroimaging,” International Journal of Imaging Systems and Technology 20, no. 1 (2010): 2–13, 10.1002/ima.20219.

[18] U. Katscher and P. Börnert , “Parallel RF Transmission in MRI,” NMR in Biomedicine 19, no. 3 (2006): 393–400, 10.1002/nbm.1049. 16705630

[19] D. Hernandez and K. N. Kim , “A Review on the RF Coil Designs and Trends for Ultra High Field Magnetic Resonance Imaging,” Investigative Magnetic Resonance Imaging. 24 (2020): 95, 10.13104/imri.2020.24.3.95.

[20] O. Kraff and H. H. Quick , “Radiofrequency Coils for 7 Tesla MRI,” Topics in Magnetic Resonance Imaging : TMRI. 28, no. 3 (2019): 145–158, 10.1097/RMR.0000000000000206. 31188273

[21] N. I. Avdievich , G. Solomakha , L. Ruhm , A. V. Nikulin , A. W. Magill , and K. Scheffler , “Folded‐End Dipole Transceiver Array for Human Whole‐Brain Imaging at 7 T,” NMR in Biomedicine 34, no. 8 (2021): e4541, 10.1002/nbm.4541. 33978270

[22] A. J. E. Raaijmakers , M. Italiaander , I. J. Voogt , et al., “The Fractionated Dipole Antenna: A New Antenna for Body Imaging at 7 Tesla,” Magnetic Resonance in Medicine 75, no. 3 (2016): 1366–1374, 10.1002/mrm.25596. 25939890

[23] B. Steensma , P. F. van de Moortele , A. Ertürk , et al., “Introduction of the Snake Antenna Array: Geometry Optimization of a Sinusoidal Dipole Antenna for 10.5T Body Imaging With Lower Peak SAR,” Magnetic Resonance in Medicine 84, no. 5 (2020): 2885–2896, 10.1002/mrm.28297. 32367560 PMC7496175

[24] B. R. Steensma , S. Zijlema , A. J. E. Raaijmakers , C. C. van Leeuwen , and C. A. T. van den Berg , “The (Un)expected Benefits of Coaxial Antennas for MRI,” in *2021 International Conference on Electromagnetics in Advanced Applications (ICEAA)*, (2021): 344–348.

[25] M. K. Woo , L. Delabarre , M. Waks , et al., “Comparison of 16‐Channel Asymmetric Sleeve Antenna and Dipole Antenna Transceiver Arrays at 10.5 Tesla MRI,” IEEE Transactions on Medical Imaging 40, no. 4 (2021): 1147–1156, 10.1109/TMI.2020.3047354. 33360987 PMC8078892

[26] I. Zivkovic and Castro dCA, Webb A., “Design and Characterization of an Eight‐Element Passively Fed Meander‐Dipole Array With Improved Specific Absorption Rate Efficiency for 7T Body Imaging,” NMR in Biomedicine 32, no. 8 (2019): e4106, 10.1002/nbm.4106. 31131944 PMC6771742

[27] S. Gunamony , M. Kozlov , J. Hoffmann , R. Turner , K. Scheffler , and R. Pohmann , “A 16‐Channel Dual‐Row Transmit Array in Combination With a 31‐Element Receive Array for Human Brain Imaging at 9.4 T,” Magnetic Resonance in Medicine 71, no. 2 (2014): 870–879, 10.1002/mrm.24726. 23483645

[28] X. Li , J. W. Pan , N. I. Avdievich , H. P. Hetherington , and J. V. Rispoli , “Electromagnetic Simulation of a 16‐Channel Head Transceiver at 7 T Using Circuit‐Spatial Optimization,” Magnetic Resonance in Medicine 85, no. 6 (2021): 3463–3478, 10.1002/mrm.28672. 33533500 PMC8124020

[29] G. Adriany , P. F. van de Moortele , F. Wiesinger , et al., “Transmit and Receive Transmission Line Arrays for 7 Tesla Parallel Imaging,” Magnetic Resonance in Medicine 53, no. 2 (2005): 434–445, 10.1002/mrm.20321. 15678527

[30] S. Orzada , K. Solbach , M. Gratz , et al., “A 32‐Channel Parallel Transmit System Add‐On for 7T MRI,” PLoS ONE 14, no. 9 (2019): 1–20, 10.1371/journal.pone.0222452. PMC6742215 31513637

[31] A. Andreychenko , H. Kroeze , D. W. J. Klomp , J. J. W. Lagendijk , P. R. Luijten , and C. A. T. van den Berg , “Coaxial Waveguide for Travelling Wave MRI at Ultrahigh Fields,” Magnetic Resonance in Medicine 70, no. 3 (2013): 875–884, 10.1002/mrm.24496. 23023780

[32] A. Destruel , J. Jin , E. Weber , et al., “Integrated Multi‐Modal Antenna With Coupled Radiating Structures (I‐MARS) for 7T pTx Body MRI,” IEEE Transactions on Medical Imaging 41, no. 1 (2022): 39–51, 10.1109/TMI.2021.3103654. 34370662

[33] T. Santini , S. Wood , N. Krishnamurthy , T. Martins , H. J. Aizenstein , and T. S. Ibrahim , “Improved 7 Tesla Transmit Field Homogeneity With Reduced Electromagnetic Power Deposition Using Coupled Tic Tac Toe Antennas,” Scientific Reports 11, no. 1 (2021): 3370, 10.1038/s41598-020-79807-9. 33564013 PMC7873125

[34] G. Solomakha , J. T. Svejda , C. van Leeuwen , et al., “A Self‐Matched Leaky‐Wave Antenna for Ultrahigh‐Field Magnetic Resonance Imaging With low Specific Absorption Rate,” Nature Communications 12, no. 1 (2021): 455, 10.1038/s41467-020-20708-w. PMC7815766 33469005

[35] D. M. Hudson , C. Heales , and R. Meertens , “Review of Claustrophobia Incidence in MRI: A Service Evaluation of Current Rates Across a Multi‐Centre Service,” Radiography 28, no. 3 (2022): 780–787, 10.1016/j.radi.2022.02.010. 35279401

[36] M. C. Meyer , R. Scheeringa , A. G. Webb , N. Petridou , O. Kraff , and D. G. Norris , “Adapted Cabling of an EEG Cap Improves Simultaneous Measurement of EEG and fMRI at 7T,” Journal of Neuroscience Methods 331 (2020): 108518, 10.1016/j.jneumeth.2019.108518. 31734326

[37] E. J. Allen , G. St‐Yves , Y. Wu , et al., “A Massive 7T fMRI Dataset to Bridge Cognitive Neuroscience and Artificial Intelligence,” Nature Neuroscience 25, no. 1 (2022): 116–126, 10.1038/s41593-021-00962-x. 34916659

[38] N. C. Benson , K. W. Jamison , M. J. Arcaro , et al., “The Human Connectome Project 7 Tesla Retinotopy Dataset: Description and Population Receptive Field Analysis,” Journal of Vision 18, no. 13 (2018): 23, 10.1167/18.13.23. PMC6314247 30593068

[39] A. K. Schobert , C. Corradi‐Dell’Acqua , S. Frühholz , W. van der Zwaag , and P. Vuilleumier , “Functional Organization of Face Processing in the Human Superior Temporal Sulcus: A 7T High‐Resolution fMRI Study,” Social Cognitive and Affective Neuroscience 13, no. 1 (2017): 102–113, 10.1093/scan/nsx119. PMC5793830 29140527

[40] S. Da Costa , J. Clément , R. Gruetter , and Ö. Ipek , “Evaluation of the Whole Auditory Pathway Using High‐Resolution and Functional MRI at 7T Parallel‐Transmit,” PLoS ONE 16, no. 9 (2021): 1–15, 10.1371/journal.pone.0254378. PMC8423236 34492032

[41] L. K. Faes and Martino dF, Huber L., “Cerebral Blood Volume Sensitive Layer‐fMRI in the Human Auditory Cortex at 7T: Challenges and Capabilities,” PLoS ONE 18, no. 2 (2023): 1–22, 10.1371/journal.pone.0280855. PMC9910709 36758009

[42] G. Batsikadze , N. Diekmann , T. M. Ernst , et al., “The Cerebellum Contributes to Context‐Effects During Fear Extinction Learning: A 7T fMRI Study,” NeuroImage 253 (2022): 119080, 10.1016/j.neuroimage.2022.119080. 35276369

[43] J. Wirsich , J. Jorge , G. R. Iannotti , et al., “The Relationship Between EEG and fMRI Connectomes Is Reproducible Across Simultaneous EEG‐fMRI Studies From 1.5T to 7T,” NeuroImage 231 (2021): 117864, 10.1016/j.neuroimage.2021.117864. 33592241

[44] Y. Donoshita , U. S. Choi , H. Ban , and I. Kida , “Assessment of Olfactory Information in the Human Brain Using 7‐Tesla Functional Magnetic Resonance Imaging,” NeuroImage 236 (2021): 118212, 10.1016/j.neuroimage.2021.118212. 34082117

[45] X. Miao , A. G. Paez , S. Rajan , et al., “Functional Activities Detected in the Olfactory Bulb and Associated Olfactory Regions in the Human Brain Using T2‐Prepared BOLD Functional MRI at 7T,” Frontiers in Neuroscience 15 (2021): 1–17, 10.3389/fnins.2021.723441. PMC8476065 34588949

[46] M. Akselrod , R. Martuzzi , W. van der Zwaag , O. Blanke , and A. Serino , “Relation Between Palm and Finger Cortical Representations in Primary Somatosensory Cortex: A 7T fMRI Study,” Human Brain Mapping 42, no. 7 (2021): 2262–2277, 10.1002/hbm.25365. 33621380 PMC8046155

[47] C. Chang , D. A. Leopold , M. L. Schölvinck , et al., “Tracking Brain Arousal Fluctuations With fMRI,” National Academy of Sciences of the United States of America 113, no. 16 (2016): 4518–4523, 10.1073/pnas.1520613113. PMC4843437 27051064

[48] G. Wyssen , M. Morrison , A. Korda , et al., “Measuring the Influence of Magnetic Vestibular Stimulation on Nystagmus, Self‐Motion Perception, and Cognitive Performance in a 7T MRT,” JoVE. 193 (2023): e64022, 10.3791/64022. 36939227

[49] M. Haeberlin , L. Kasper , C. Barmet , et al., “Real‐Time Motion Correction Using Gradient Tones and Head‐Mounted NMR Field Probes,” Magnetic Resonance in Medicine 74, no. 3 (2015): 647–660, 10.1002/mrm.25432. 25219482

[50] J. Schulz , T. Siegert , E. Reimer , et al., “An Embedded Optical Tracking System for Motion‐Corrected Magnetic Resonance Imaging at 7T,” Magnetic Resonance Materials in Physics, Biology and Medicine 25, no. 6 (2012): 443–453, 10.1007/s10334-012-0320-0. 22695771

[51] D. Stucht , K. A. Danishad , P. Schulze , F. Godenschweger , M. Zaitsev , and O. Speck , “Highest Resolution In Vivo Human Brain MRI Using Prospective Motion Correction,” PLoS ONE 10, no. 7 (2015): 1–17, 10.1371/journal.pone.0133921. PMC4520483 26226146

[52] C. H. Choi , A. Webb , S. Orzada , M. Kelenjeridze , N. J. Shah , and J. Felder , “A Review of Parallel Transmit Arrays for Ultra‐High Field MR Imaging,” IEEE Reviews in Biomedical Engineering (2023): 1–19, 10.1109/RBME.2023.3244132. 37022919

[53] S. H. G. Rietsch , H. H. Quick , and S. Orzada , “Impact of Different Meander Sizes on the RF Transmit Performance and Coupling of Microstrip Line Elements at 7 T,” Medical Physics 42, no. 8 (2015): 4542–4552, 10.1118/1.4923177. 26233183

[54] A. J. E. Raaijmakers , P. R. Luijten , and C. A. T. van den Berg , “Dipole Antennas for Ultrahigh‐Field Body Imaging: A Comparison With Loop Coils,” NMR in Biomedicine 29, no. 9 (2016): 1122–1130, 10.1002/nbm.3356. 26278544

[55] J. D. Clément , R. Gruetter , and Ö. Ipek , “A Human Cerebral and Cerebellar 8‐Channel Transceive RF Dipole Coil Array at 7T,” Magnetic Resonance in Medicine 81, no. 2 (2019): 1447–1458, 10.1002/mrm.27476. 30226637

[56] Z. Cao , X. Yan , J. C. Gore , and W. A. Grissom , “Designing Parallel Transmit Head Coil Arrays Based on Radiofrequency Pulse Performance,” Magnetic Resonance in Medicine 83, no. 6 (2020): 2331–2342, 10.1002/mrm.28068. 31722120 PMC7047538

[57] L. Winter , C. Özerdem , W. Hoffmann , et al., “Design and Evaluation of a Hybrid Radiofrequency Applicator for Magnetic Resonance Imaging and RF Induced Hyperthermia: Electromagnetic Field Simulations up to 14.0 Tesla and Proof‐of‐Concept at 7.0 Tesla,” PLoS ONE 8, no. 4 (2013): 1–12, 10.1371/journal.pone.0061661. PMC3632575 23613896

[58] B. Steensma , A. V. Obando Andrade , D. Klomp , N. van den Berg , P. Luijten , and A. Raaijmaakers , “Body Imaging at 7 Tesla With Much Lower SAR Levels: An Introduction of the Snake Antenna Array,” in *Proceedings of the 24th Annual Meeting of the ISMRM*, (2016): 395.

[59] M. J. Hubmann , R. Kowal , S. Orzada , et al., “Simulation and Comparison of Transmit Elements for 7T Head‐Imaging with a Large Diameter Transmit Coil,” in *In Proceedings of the 31st Annual Meeting of the ISMRM*, (2023): 4583.

[60] M. J. Hubmann , R. Kowal , S. Orzada , O. Speck , and H. Maune . “Simulation of the Transmit Performance of 8 Channel Arrays for 7T Head‐Imaging With a Large Diameter Transmit Coil,” in *Proceedings of the 40th Annual Scientific Meeting of the ESMRMB, Barcelona, Spain*, (2024): 379.

[61] R. W. Brown , Y. C. N. Cheng , E. M. Haacke , M. R. Thompson , and R. Venkatesan , *Magnetic Resonance Imaging: Physical Principles and Sequence Design* , 2nd ed. (John Wiley & Sons, 2014).

[62] A. D. Yaghjian and S. R. Best , “Impedance, Bandwidth, and Q of Antennas,” IEEE Transactions on Antennas and Propagation 53, no. 4 (2005): 1298–1324, 10.1109/TAP.2005.844443.

[63] K. M. Bushby , T. Cole , J. N. Matthews , and J. A. Goodship , “Centiles for Adult Head Circumference,” Archives of Disease in Childhood 67, no. 10 (1992): 1286–1287, 10.1136/adc.67.10.1286. 1444530 PMC1793909

[64] A. Nguyen , A. Simard‐Meilleur , C. Berthiaume , R. Godbout , and L. Mottron , “Head Circumference in Canadian Male Adults: Development of a Normalized Chart,” International Journal of Morphology 30, no. 4 (2012): 1474–1480.

[65] M. N. Voelker , O. Kraff , E. Pracht , et al., “Quality Assurance Phantoms and Procedures for UHF MRI—The German Ultrahigh Field Imaging (GUFI) Approach,” in *Proceedings of the 25th Annual Meeting of the ISMRM*, (2017): 3912.

[66] A. Kuehne , S. Goluch , P. Waxmann , et al., “Power Balance and Loss Mechanism Analysis in RF Transmit Coil Arrays,” Magnetic Resonance in Medicine 74, no. 4 (2015): 1165–1176, 10.1002/mrm.25493. 25324179

[67] J. K. Stelter , M. E. Ladd , and T. M. Fiedler , “Numerical Comparison of Local Transceiver Arrays of Fractionated Dipoles and Microstrip Antennas for Body Imaging at 7T,” NMR in Biomedicine 35, no. 8 (2022): e4722, 10.1002/nbm.4722. 35226966

[68] B. Guérin , M. Gebhardt , S. Cauley , E. Adalsteinsson , and L. L. Wald , “Local Specific Absorption Rate (SAR), Global SAR, Transmitter Power, and Excitation Accuracy Trade‐Offs in Low Flip‐Angle Parallel Transmit Pulse Design,” Magnetic Resonance in Medicine 71, no. 4 (2014): 1446–1457, 10.1002/mrm.24800. 23776100 PMC3871989

[69] IEC 60601‐2‐33:2022 , *Medical Electrical Equipment—Part 2‐33: Particular Requirements for the Basic Safety and Essential Performance of Magnetic Resonance Equipment for Medical Diagnosis. Standard* (International Electrotechnical Commission, 2022).

[70] Z. Wang , J. C. Lin , W. Mao , W. Liu , M. B. Smith , and C. M. Collins , “SAR and Temperature: Simulations and Comparison to Regulatory Limits for MRI,” Journal of Magnetic Resonance Imaging: JMRI 26, no. 2 (2007): 437–441, 10.1002/jmri.20977. 17654736 PMC4040525

[71] S. Wolf , D. Diehl , M. Gebhardt , J. Mallow , and O. Speck , “SAR Simulations for High‐Field MRI: How Much Detail, Effort, and Accuracy Is Needed?,” Magnetic Resonance in Medicine 69, no. 4 (2013): 1157–1168, 10.1002/mrm.24329. 22611018

[72] IEC/IEEE 62704‐1:2017 , *Determining the Peak Spatial? Average Specific Absorption Rate (SAR) in the Human Body From Wireless Communications Devices, 30 MHz to 6 GHz—Part 1: General Requirements for Using the Finite Difference Time‐Domain (FDTD) Method for SAR Calculations* (Standard, International Electrotechnical Commission, 2017).

[73] V. L. Yarnykh , “Actual Flip‐Angle Imaging in the Pulsed Steady State: A Method for Rapid Three‐Dimensional Mapping of the Transmitted Radiofrequency Field,” Magnetic Resonance in Medicine 57, no. 1 (2007): 192–200, 10.1002/mrm.21120. 17191242

[74] J. Clément , R. Gruetter , and Ö. Ipek , “A Combined 32‐Channel Receive‐Loops/8‐Channel Transmit‐Dipoles Coil Array for Whole‐Brain MR Imaging at 7T,” Magnetic Resonance in Medicine 82, no. 3 (2019): 1229–1241, 10.1002/mrm.27808. 31081176 PMC6618274

[75] J. T. Vaughan and J. R. Griffiths , eds., “ *RF Coils for MRI* ,” in *EMR Handbooks* , 1st ed. (Wiley, 2012).

[76] S. H. G. Rietsch , S. Orzada , A. K. Bitz , M. Gratz , M. E. Ladd , and H. H. Quick , “Parallel Transmit Capability of Various RF Transmit Elements and Arrays at 7T MRI,” Magnetic Resonance in Medicine 79, no. 2 (2018): 1116–1126, 10.1002/mrm.26704. 28394080

[77] S. Orzada , T. M. Fiedler , and M. E. Ladd , “Hybrid Algorithms for SAR Matrix Compression and the Impact of Post‐Processing on SAR Calculation Complexity,” Magnetic Resonance in Medicine (2024): 1–11, 10.1002/mrm.30235. 39056341

[78] B. Guérin , M. Gebhardt , P. Serano , et al., “Comparison of Simulated Parallel Transmit Body Arrays at 3 T Using Excitation Uniformity, Global SAR, Local SAR, and Power Efficiency Metrics,” Magnetic Resonance in Medicine 73, no. 3 (2015): 1137–1150, 10.1002/mrm.25243. 24752979 PMC4201892

[79] F. Breuer , M. Blaimer , M. Mueller , R. Heidemann , M. Griswold , and J. Peter , “The Use of Principal Component Analysis (PCA) for Estimation of the Maximum Reduction Factor In 2D Parallel Imaging,” in *Proceedings of the 13th Annual Meeting of the ISMRM*, (2014): 2668.

[80] T. M. Fiedler , S. Orzada , M. Flöser , et al., “Performance Analysis of Integrated RF Microstrip Transmit Antenna Arrays With High Channel Count for Body Imaging at 7 T,” NMR in Biomedicine 34, no. 7 (2021): e4515, 10.1002/nbm.4515. 33942938

[81] B. Gruber , M. Froeling , T. Leiner , and D. W. J. Klomp , “RF Coils: A Practical Guide for Nonphysicists,” Journal of Magnetic Resonance Imaging: JMRI. 48, no. 3 (2018): 590–604, 10.1002/jmri.26187. 29897651 PMC6175221

[82] N. I. Avdievich , J. W. Pan , and H. P. Hetherington , “Resonant Inductive Decoupling (RID) for Transceiver Arrays to Compensate for Both Reactive and Resistive Components of the Mutual Impedance,” NMR in Biomedicine 26, no. 11 (2013): 1547–1554, 10.1002/nbm.2989. 23775840 PMC3800502

[83] D. Brizi , N. Fontana , and A. Monorchio , “Analytical Approach for MRI RF Array Coils Decoupling by Using Counter‐Coupled Passive Resonators,” IEEE Open Journal of Antennas and Propagation 2 (2021): 249–258, 10.1109/OJAP.2021.3059495.

[84] I. R. O. Connell , K. M. Gilbert , M. A. Abou‐Khousa , and R. S. Menon , “MRI RF Array Decoupling Method With Magnetic Wall Distributed Filters,” IEEE Transactions on Medical Imaging 34, no. 4 (2015): 825–835, 10.1109/TMI.2014.2378695. 25838388

[85] N. I. Avdievich , G. Solomakha , L. Ruhm , A. Henning , and K. Scheffler , “Unshielded Bent Folded‐End Dipole 9.4 T Human Head Transceiver Array Decoupled Using Modified Passive Dipoles,” Magnetic Resonance in Medicine 86, no. 1 (2021): 581–597, 10.1002/mrm.28711. 33629436

[86] A. Hurshkainen , M. S. M. Mollaei , M. Dubois , et al., “Decoupling of Closely Spaced Dipole Antennas for Ultrahigh Field MRI With Metasurfaces,” IEEE Transactions on Antennas and Propagation 69, no. 2 (2021): 1094–1106, 10.1109/TAP.2020.3016495.

[87] X. Yan , X. Zhang , L. Wei , and R. Xue , “Design and Test of Magnetic Wall Decoupling for Dipole Transmit/Receive Array for MR Imaging at the Ultrahigh Field of 7T,” Applied Magnetic Resonance 46, no. 1 (2015): 59–66, 10.1007/s00723-014-0612-9. 28955135 PMC5612434

[88] A. J. E. Raaijmakers , O. Ipek , D. W. J. Klomp , et al., “Design of a Radiative Surface Coil Array Element at 7 T: The Single‐Side Adapted Dipole Antenna,” Magnetic Resonance in Medicine 66, no. 5 (2011): 1488–1497, 10.1002/mrm.22886. 21630342

[89] A. Sadeghi‐Tarakameh , B. Khalichi , X. Wu , G. Metzger , and Y. Eryaman . “Non‐Uniform Dielectric Substrate (NODES) Antenna Design for Cardiac Imaging at 7T,” in *In Proceedings of the 29th Annual Meeting of the ISMRM*, online, (2011), 1398.

[90] D. Wenz and R. Gruetter , “Dipole‐Fed Rectangular Dielectric Resonator Antennas for Magnetic Resonance Imaging at 7 T: The Impact of Quasi‐Transverse Electric Modes on Transmit Field Distribution,” Frontiers in Physics 9 (2021): 675509, 10.3389/fphy.2021.675509.

[91] S. Schmidt , M. A. Ertürk , X. He , T. Haluptzok , Y. Eryaman , and G. J. Metzger , “Improved 1H Body Imaging at 10.5 T: Validation and VOP‐Enabled Imaging In Vivo With a 16‐Channel Transceiver Dipole Array,” Magnetic Resonance in Medicine 91, no. 2 (2024): 513–529, 10.1002/mrm.29866. 37705412 PMC10850915

[92] N. I. Avdievich , G. Solomakha , L. Ruhm , K. Scheffler , and A. Henning , “Decoupling of Folded‐End Dipole Antenna Elements of a 9.4 T Human Head Array Using an RF Shield,” NMR in Biomedicine 33, no. 9 (2020): e4351, 10.1002/nbm.4351. 32618047

