# Supplementary material for: Towards Large Diameter Transmit Coils for 7‐T Head Imaging: A Detailed Comparison of a Set of Transmit Element Design Concepts
Source: NMR Biomed. 2025 Apr 5;38(5):e70030. doi: 10.1002/nbm.70030 (PMC11971727; doi:10.1002/nbm.70030)
Supplement: Supplementary file 1 — Supporting_Information.pdf [file NBM-38-e70030-s001.pdf]

Supporting Information

Further description of the Tx elements

The used transmit elements were taken from the literature and developed for different purposes. The following supporting table ST 1 gives a summary on the advantages and disadvantages of the corresponding Tx elements taken from the original publications as well as their target regions and application. The loop-based elements were added since they can be considered as the gold standard for 7T head imaging.

Supporting Table ST 1: Summary of the advantages and disadvantages as well as target regions and applications of the evaluated Tx elements taken from the literature.

Transmit element	Advantages	Disadvantages	Target Region
Folded End Dipole[21]	- Higher power efficiency compared to commercial loop array<br>- Better whole brain coverage	- Non-ideal SNR since no local Rx coil in use<br>- So far, a tight-fitting array	Head/brain
Microstrip Meander Element[62]	- Very good decoupling<br>- Optimal H-field distribution	- Lower SAR efficiency compared to dipoles	Body
Fractionated Dipole[63]	- Lower SAR levels compared to classical dipoles	- Lower receive performance compared to classical dipoles	Body/Prostate
Regular Dipole[64]	- Higher power efficiency with moderate SAR	- Tight-fitting array	Head/Brain/Cerebell-um
Rectangular Loop[6]	-	-	Head/Brain
Circular Loop[54]	-	-	Head/Brain
Passively Fed Dipole[26]	- Lower SAR compared to fractionated dipole	- Lower power efficiency	Body/Prostate
Bowtie Antenna[65]	- High power efficiency	- Low SAR efficiency	Brain/Thermo Ablation
Snake Antenna[66]	- Higher SAR efficiency compared to the fractionated dipole	- Lower power efficiency	Body

Simulation Setup and ROIs

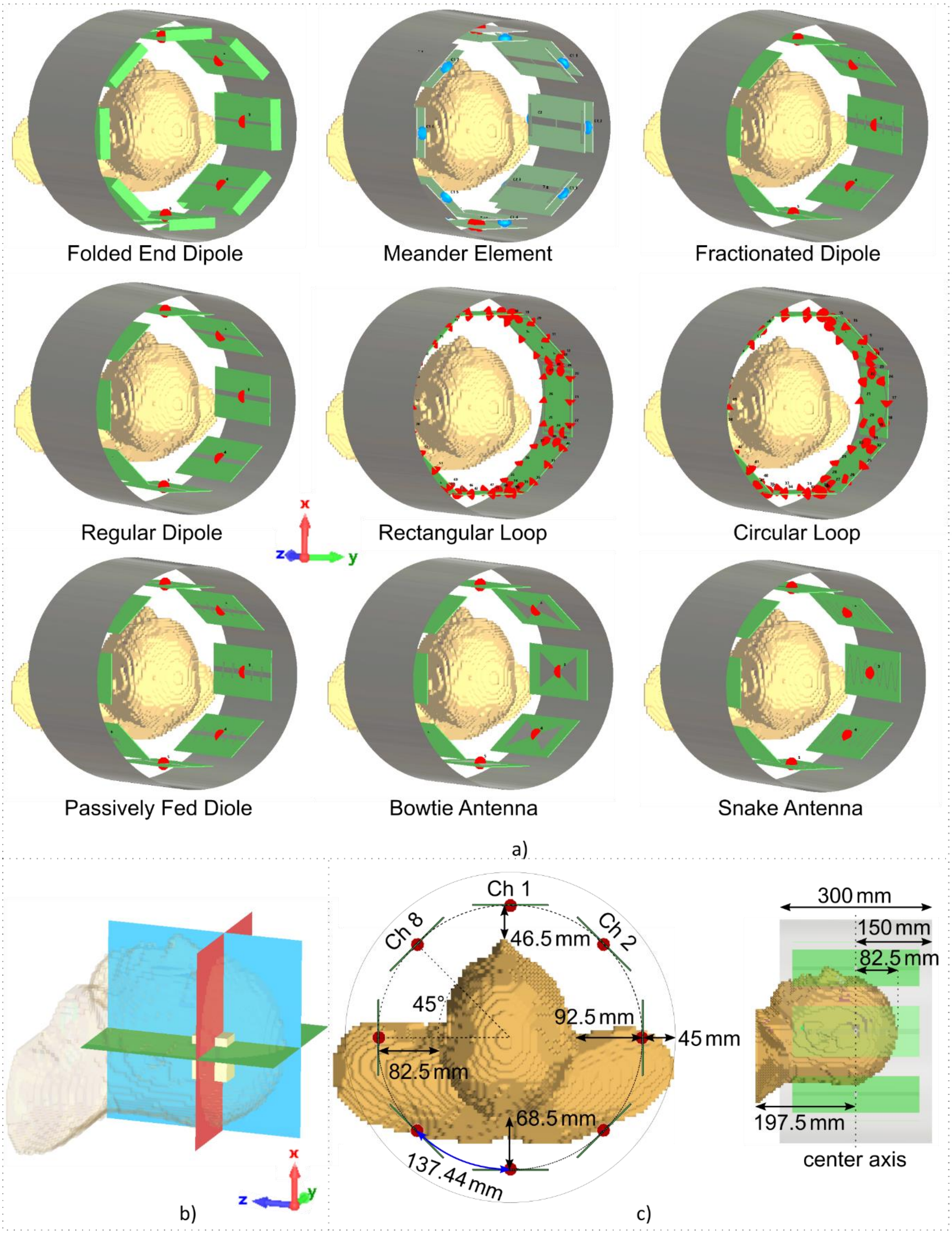

Supporting Figure S 1 Simulation Setups for the nine different Tx elements described in Section 2.1 as an addition to Figure 3 in (a). In (b) the described regions of interest (ROIs) for the voxel-wise power and SAR efficiencies and the L-curve calculation. Red shows the transversal slice, blue the sagittal slice and green the coronal slice. The yellow box shows the ROI for the hippocampus as described in Figure 3d with the blue rectangle. In (c) additional information about the geometric arrangement of the eight-channel configuration are provided.

Decoupling of Loops

In this study, capacitive decoupling was used for the loop-based Tx elements. Therefore, two decoupling capacitors were placed in between adjacent loops (e.g. C_{dec21}). This enables a current from one element to the other which can be controlled by the size of the capacitors. If the decoupling capacitors are properly dimensioned, this current is equal in amplitude but in opposite phase to the current induced through the elements magnetic flux. These two currents neutralize each other and thereby reduce the coupling. The decoupling was performed in CST Microwave Studio 2022 co-simulation using lumped element capacitors. Supporting Figure S2a) and b) show where the decoupling capacitors were placed. In Supporting Figure S2c) a section of the CST co-simulation is depicted to exemplarily show, how the connection was actually done.

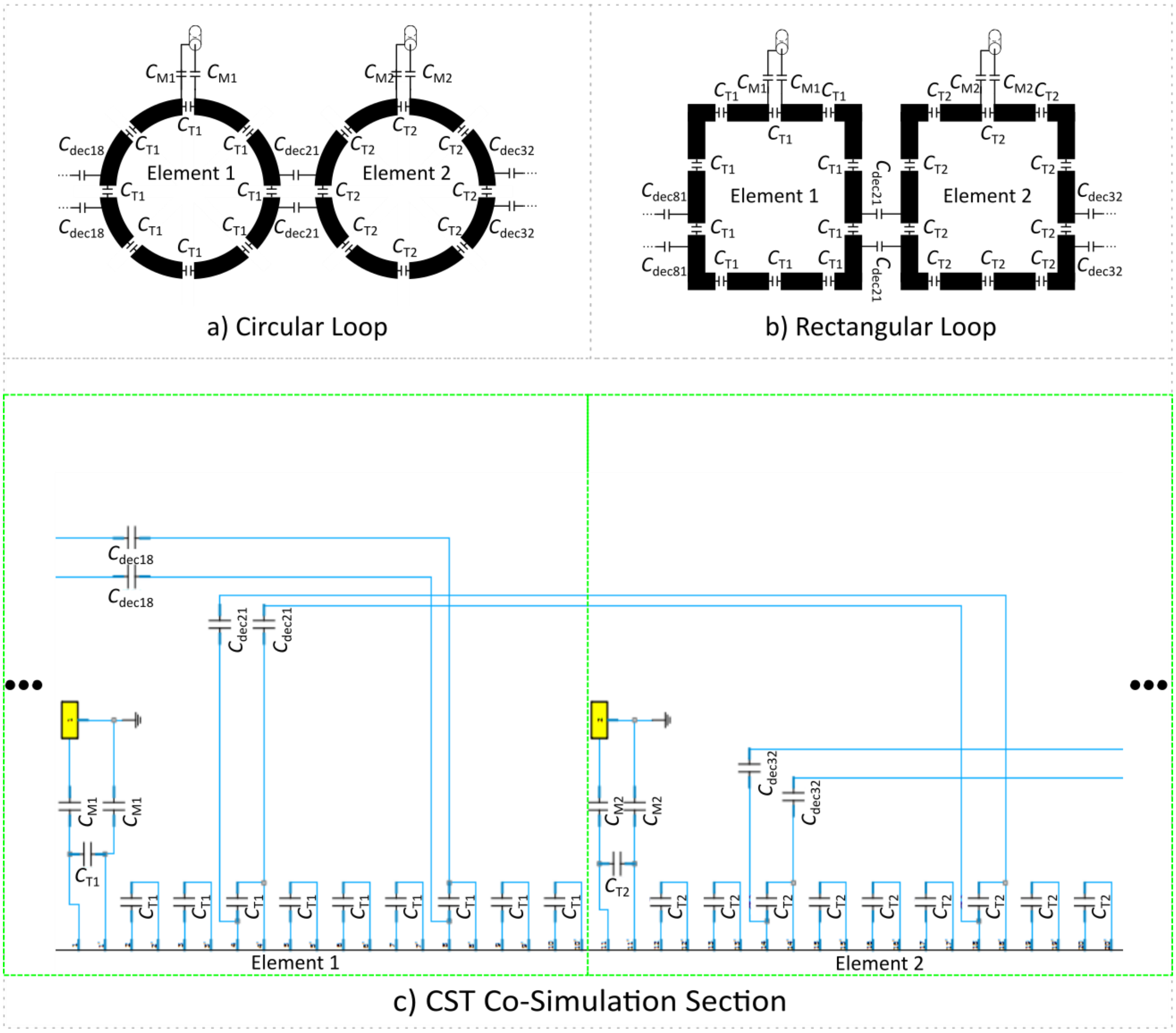

Supporting Figure S 2 Placement of the decoupling capacitors for the circular (a) and the rectangular (b) loop depicted for the first two elements. The same pattern was retained for the remaining elements. In (c) the schematic of the CST Co-Simulation is depicted for two elements of the rectangular loop.

Loading Dependence

The loading dependence of the nine Tx elements was evaluated in simulation and in measurements as described in Section 2.2.1. The results depicted in Figure 4 are based on the S-parameter results shown in Supporting Figure S3.

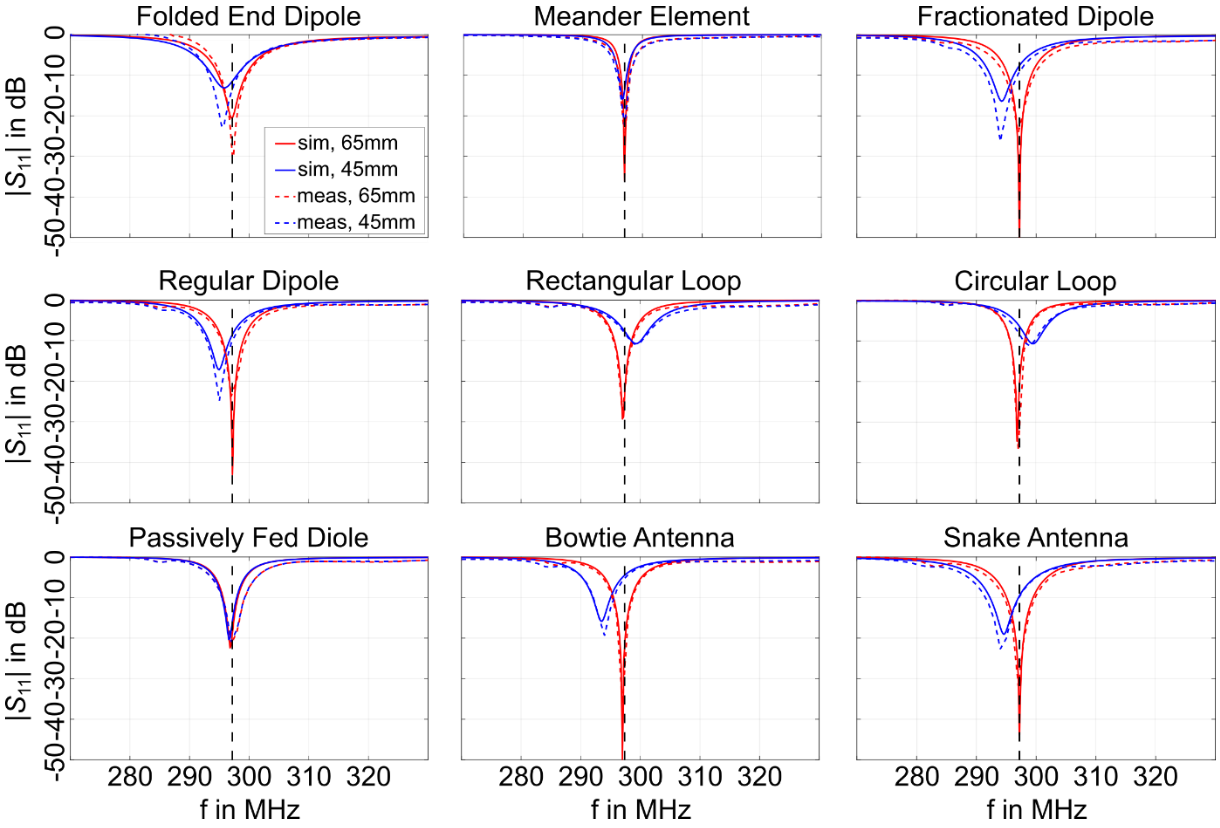

Supporting Figure S 3 S-parameter plots of the input reflection $|S_{11}|$ for the nine Tx elements. The measured data (meas) is represented by the dashed lines and the solid lines represent the simulated data (sim). The red curves (65 mm) represent the tuned state whereby the blue curves (45 mm) represent the data after the decrease in distance to 45 mm.

Power Efficiency Deviation Examination

Since there was a strong discrepancy between the power efficiencies using the head of the Hugo voxel model and using the GUFI-phantom, an additional examination was performed using CST Microwave Studio 2022 and a head like phantom with material properties equal to the GUFI-phantom for the circular loop and the fractionated dipole, see Supporting Figure S4. The simulation setup is depicted in Supporting Figure S4a) and b). The three setups were compared towards their power efficiency dependence on the phantom geometry using $|B_1^+|$ -maps and two dedicated points marked in Supporting Figure S4c).

The results show that the power efficiency at the two marked points increased for the circular loop and decreased for the fractionated dipole, when the load was changed towards the Hugo voxel model through the head-shaped GUFI-phantom. Furthermore, the absolute change in power efficiency for the circular loop was higher compared to the fractionated dipole. However, the field distortion due to the different loads was comparable.

It can be concluded, that the difference in power efficiency between the Tx elements using the head of the Hugo voxel model and the GUFI-phantom was based on the geometric difference.

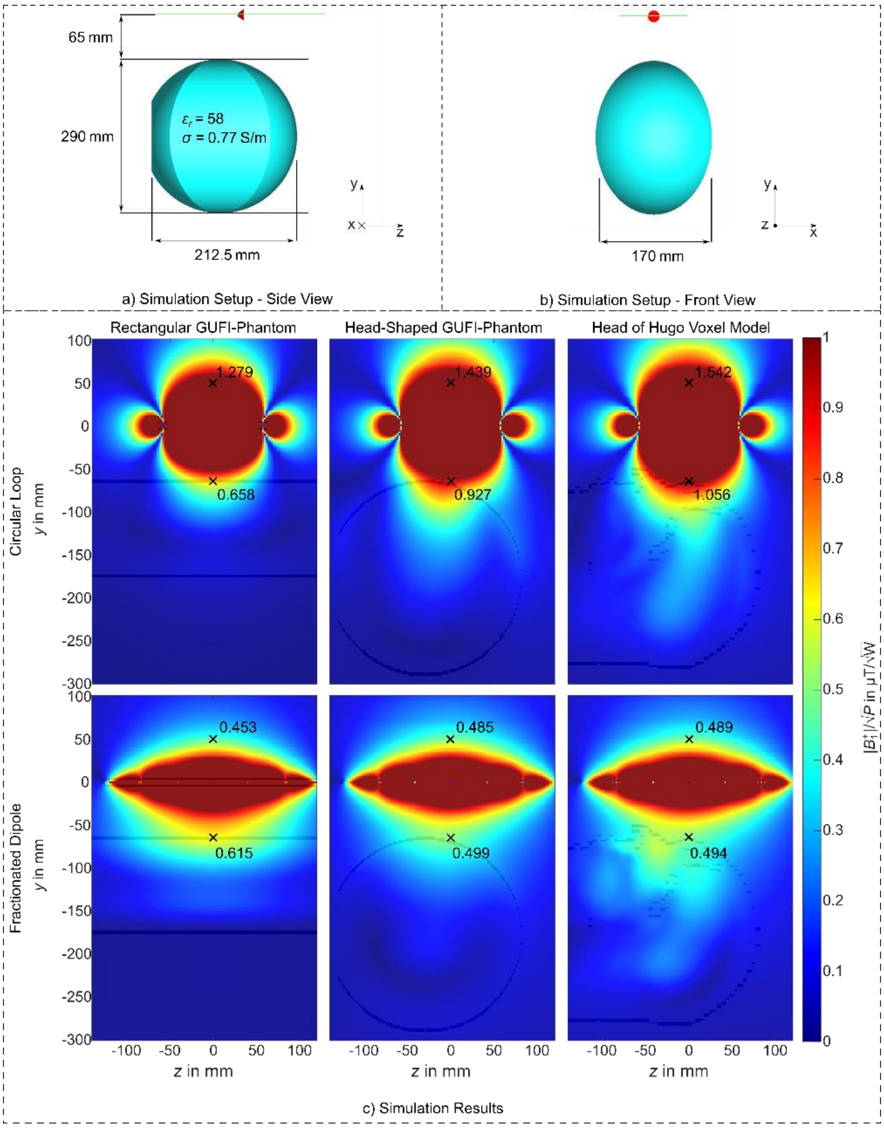

Supporting Figure S 4 Evaluation of the geometry dependence of the circular loop and the fractionated dipole; (a) Side view of the simulation model and setup for the evaluation of the head shaped GUFI-phantom exemplary for the fractionated dipole with the feed port in red and the substrate in green; (b) Front view of the head shaped GUFI-

Phantom; (c) Simulation results of the geometry dependence analysis for the rectangular-shaped GUFI-phantom (see Section 2.2.4), the head-shaped GUFI-phantom and the head of the Hugo voxel model (see Section 2.2).

Power Efficiency of a larger Rectangular Loop

For the loop-based elements a 125mm times 115mm rectangular loop was chosen. However, the dimensions of the loops have many degrees of freedom. Therefore, a larger rectangular loop might be used to get dipole-like dimensions in the z direction. To make a statement about this effect on the described application a larger rectangular loop was simulated (Supporting Figure S5a)).

The results show that the power efficiency as well as the SAR efficiency for the single element simulation as well as for the 8 channel arrays slightly decreased using a larger loop compared to the smaller rectangular loop. However, it is still considerably higher than for the dipole-based elements. The SnSV for the large rectangular loop was calculated to 4.27, which is similar to the rectangular loop and higher than for the dipole-based Tx elements.

It can be concluded, that even using larger loops will not alter the statement, that for larger diameter transmit arrays for head imaging at 7T, loop-based elements are the preferable element type.

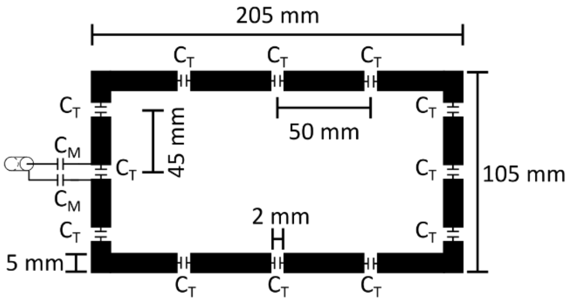

a) Long Rectangular Loop

		Power Efficiency in µT/√W/kg		SAR Efficiency in µT/√W/kg	
		65mm	165mm	65mm	165mm
1 Ch	0°	0.70	0.33	1.56	0.75
	45°	0.61	0.34	1.12	0.65
	90°	0.47	0.34	0.90	0.64
8 Ch CP$^+$		0.55		1.25	

b) Power and SAR Efficiency

Supporting Figure S 5 Dimensions of the large rectangular loop in a) and results for the single channel as well as the 8-channel configuration power and SAR efficiencies. For the single channel evaluation, the power and SAR efficiency was evaluated at 2 depths as well as 3 positions of the rectangular loop towards the head of the Hugo voxel model, as described in Figure 3. The 8-channel configuration was evaluated in CP$^+$ mode only.
